# Elongation factor Tu is a multifunctional and processed moonlighting protein

**DOI:** 10.1038/s41598-017-10644-z

**Published:** 2017-09-11

**Authors:** Michael Widjaja, Kate Louise Harvey, Lisa Hagemann, Iain James Berry, Veronica Maria Jarocki, Benjamin Bernard Armando Raymond, Jessica Leigh Tacchi, Anne Gründel, Joel Ricky Steele, Matthew Paul Padula, Ian George Charles, Roger Dumke, Steven Philip Djordjevic

**Affiliations:** 1 The ithree institute, University of Technology Sydney, PO Box 123 Broadway, NSW, 2007 Australia; 2 Technische Universität Dresden, Medizinische Fakultät Carl Gustav Carus, Institut für Medizinische Mikrobiologie und Hygiene, Fetscherstrasse 74, 01307 Dresden, Germany; 3 Proteomics Core Facility, University of Technology Sydney, PO Box 123 Broadway, NSW, 2007 Australia; 4 Quadram Institute Bioscience, Norwich Research Park, Norwich, Norfolk, NR4 7UA UK

## Abstract

Many bacterial moonlighting proteins were originally described in medically, agriculturally, and commercially important members of the low G + C Firmicutes. We show Elongation factor Tu (Ef-Tu) moonlights on the surface of the human pathogens *Staphylococcus aureus* (Sa_Ef-Tu_) and *Mycoplasma pneumoniae* (Mpn_Ef-Tu_), and the porcine pathogen *Mycoplasma hyopneumoniae* (Mhp_Ef-Tu_). Ef-Tu is also a target of multiple processing events on the cell surface and these were characterised using an N-terminomics pipeline. Recombinant Mpn_Ef-Tu_ bound strongly to a diverse range of host molecules, and when bound to plasminogen, was able to convert plasminogen to plasmin in the presence of plasminogen activators. Fragments of Ef-Tu retain binding capabilities to host proteins. Bioinformatics and structural modelling studies indicate that the accumulation of positively charged amino acids in short linear motifs (SLiMs), and protein processing promote multifunctional behaviour. Codon bias engendered by an A + T rich genome may influence how positively-charged residues accumulate in SLiMs.

## Introduction

Elongation factor Thermo unstable (Ef-Tu) is one the most abundant proteins in bacteria^1, 2^. It functions as an essential and universally conserved GTPase that ensures translational accuracy by catalysing the reaction that adds the correct amino acid to a growing nascent polypeptide chain^3^. After the incoming aminoacyl-tRNA docks with the mRNA, GTPase activity induces a conformational change releasing Ef-Tu from the ribosome^3–5^. In *Escherichia coli*, Ef-Tu is comprised of three functional domains known as domain I (amino acids 1–200), domain II (amino acids 209–299) and domain III (amino acids 301–393)^6^. Domain I forms a helix structure with Rossmann fold topology, a structural motif found in proteins that bind nucleotides, while domains II and III are largely comprised of beta sheets^3, 7^. The GTP/GDP binding domains are housed in domain I, while domains I and II are needed for nucleotide exchange. Domains II and III physically adjust to form an amino acid tRNA binding site^3, 5^. Ef-Tu sequences derived from phylogenetically diverse species share considerable sequence identity and have been used to generate phylogenetic descriptions of the tree of life^8^. In eukaryotes, domain III also has a role in actin polymerisation via an actin-bundling domain^9, 10^.

Despite its highly conserved function in protein synthesis, non-canonical functions have been described for Ef-Tu in all kingdoms of life. Ef-Tu lacks a signal secretion motif yet the ability to execute moonlighting functions often requires the molecule to localise to the cell surface. Ef-Tu is a multifunctional protein in higher order eukaryotes^11–16^, parasites^17–20^, fungi^21^ and it is has been identified on the surface of a wide range of Gram positive and Gram negative pathogenic and commensal bacteria that associate with metazoan species^2, 22–29^. Bacterial Ef-Tu interacts with nucleolin^30, 31^, fibrinogen and factor H^23, 26^, plasminogen and several complement factors^26, 27, 32^, laminin^33^, CD21^34^, fibronectin^2, 33, 35, 36^, is immunogenic^37^ and adheres to the surface of Hep-2 cells^33^ underscoring the multifunctional adhesive characteristics that have been assigned to this molecule. Ef-Tu binds sulfated carbohydrate moieties found on glycolipids and sulfomucin and promotes the binding of *Lactobacillus reuteri* to mucosal surfaces indicating that Ef-Tu can interact with carbohydrates^38^. Notably, antibodies against Ef-Tu are induced during infections caused by *Staphylococcus aureus* ^39, 40^ *Mycoplasma capricolum* ^41^, *Mycoplasma ovipneumoniae* ^37^, *Chlamydia trachomatis* ^42^, *Burkholderia pseudomallei* ^43^ and *Mycoplasma hyopneumoniae* ^44^. Ef-Tu has been identified in six surfacome studies (excludes cell membrane and envelope isolations)^45–50^ performed on *S. aureus* and Ef-Tu is one of twelve proteins consistently identified in the exoproteome of *S. aureus* from patients with bacteraemia^51^. The major staphylococcal autolysin *Alt* is implicated in playing a role in secreting cytosolic proteins including Ef-Tu into the extracellular milieu^24^. Moonlighting proteins are likely to be exported via several mechanisms including within secreted extracellular vesicles^52^, during cell lysis^53^ and via association with proteins that are secreted by the Sec machinery^54^.

**Figure 1:**
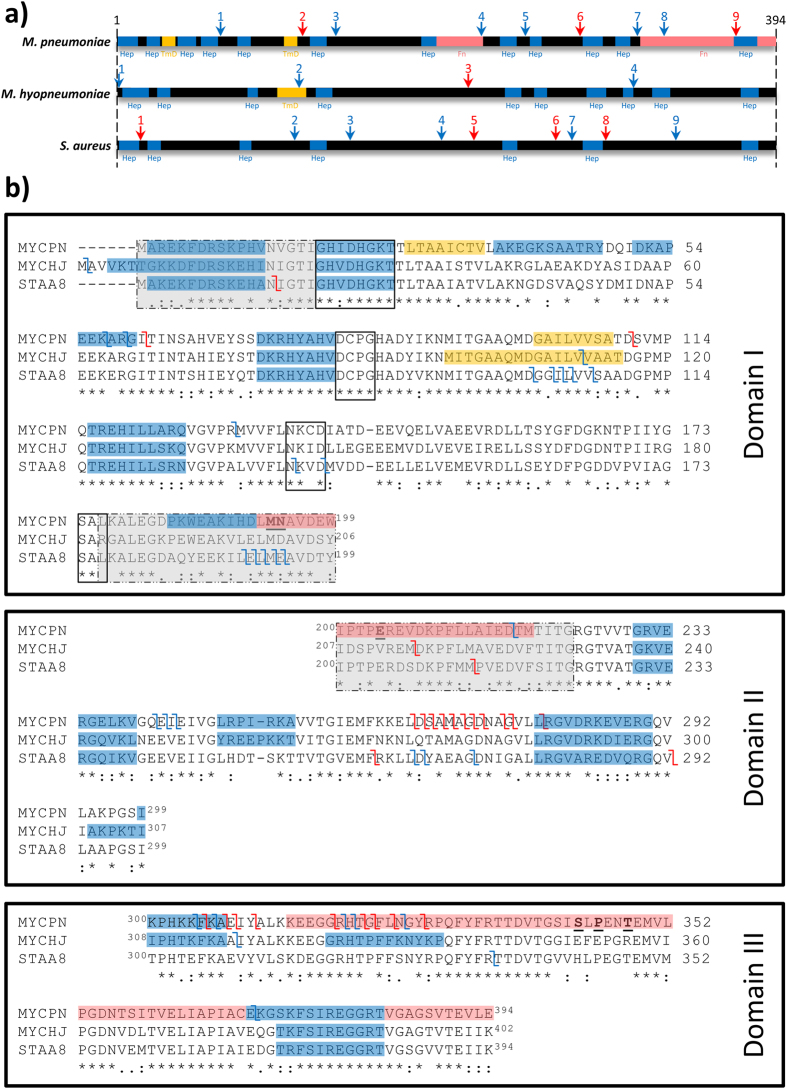
Bioinformatic analysis of Mpn_Ef-Tu_, Mhp_Ef-Tu_, and Sa_Ef-Tu_. (**a**) Schematic of Mpn_Ef-Tu_, Mhp_Ef-Tu_, and Sa_Ef-Tu_ highlighting putative heparin and fibronectin-binding domains and cleavage sites. ScanProsite^144^ was used to predict heparin-binding motifs (dark blue boxes) by searching clusters of basic residues with the “X-[HRK]-X(0,2)-[HRK]-X(0,2)-[HRK]-X” and “X-[HRK]-X(1,4)-[HRK]-X(1,4)-[HRK]-X” motifs. A putative transmembrane domain (score 505) was predicted in Mhp_Ef-Tu_ using TMpred^142^ (yellow box). In Mpn_Ef-Tu_, two fibronectin-binding regions (salmon boxes) and two predicted transmembrane domains (scores) are depicted in Panels a and b^89, 90^. Key amino acids in Mpn_Ef-Tu_ involved in binding fibronectin^89, 90^ are underlined. Cleavage sites identified in this study are shown as arrows above the black bar (blue indicates cleavage sites identified by dimethyl labelling and red indicates cleavage sites identified by the characterisation of semi-tryptic peptides by LC-MS/MS). (**b**) Amino acid sequence alignments of Mpn_Ef-Tu_, Mhp_Ef-Tu_, and Sa_Ef-Tu_. For consistency, features described in Fig. 1a are represented by the same colour scheme in Fig. 1b. Cleavage sites identified in this study are depicted by the symbol *Ζ*. Sequence alignments have been separated into the three domains and the nucleotide-binding motifs (boxed regions) and the two pattern recognition receptors (broken black outline grey box from *Acidovorax avenae* ^65^ and *Brassica*-specific receptors^64^) are shown.

The ability of Ef-Tu to be secreted onto the cell surface occurred early in the evolutionary interplay between plant pathogenic bacteria and their eukaryote hosts and is a well described pathogen associated molecular pattern (PAMP) molecule^55, 56^. Plants have evolved pattern recognition receptors (PRR) in their cell membranes that are designed specifically to recognise PAMP molecules released by bacterial and fungal pathogens^56–62^. An Ef-Tu receptor (EFR) found within Brassica lineages^63, 64^ recognises the highly conserved N-terminal 18 amino acids (elf18) in the native Ef-Tu molecule^56, 63, 64^. Binding triggers signal transduction events in plant roots that ensure that pathogenic bacteria are either contained within callose deposits, destroyed by cellular apoptosis, or succumb to an oxidative burst elicited by the production of hydrogen peroxide^63^. A region spanning surface exposed amino acids 176–225, in Ef-Tu from the Gram-negative bacterial pathogen *Acidovorax avenae*, interacts with a different PRR in monocotyledonous plants (see Fig. 1)^65^. EFR has been transferred from the Brassica species *Arabidopsis thaliana* into the monocot species, rice and transgenic rice plants display enhanced innate immune responses when exposed to elf18 from *Xanthomonas oryza*, a major rice pathogen^66^. These studies show that plants have evolved sophisticated molecular machinery to identify Ef-Tu that is released onto the cell surface by diverse plant pathogenic bacteria.

Protein cleavage is emerging as an important post-translational modification that can expand protein function^67–70^. This is evident in the genome reduced Mollicutes where species specific Mycoplasmal adhesins and lipoproteins are targets of complex processing events^67, 71–86^. Cleavage fragments are retained on the bacterial cell surface and function as adhesins that bind heparin-like glycosaminoglycans^67, 73–75, 77, 79, 80^, fibronectin^67, 76, 78, 84^ and circulatory molecules such a plasmin(ogen) that regulate the fibrinolytic system^67, 76, 78, 79, 81^. Cleavage motifs have been chemically defined in *M. hyopneumoniae* using mass spectrometry and occur at phenylalanine residues in the motif S/T-X-F↓-X-D/E, within stretches of hydrophobic amino acids, and at trypsin-like sites in diverse molecules including adhesins, lipoproteins and in metabolic enzymes that traffic to the cell surface^77, 79, 82, 83, 85^. Cleavage fragments are known to be further processed by aminopeptidases^83, 85^ that also localise on the cell surface^87, 88^. We propose that protein processing represents another layer by which proteins can expand and modify protein function and is under recognised as a post-translational modification in prokaryotes.

In this study we identified Ef-Tu, and an extensive repertoire of processed cleavage fragments of Ef-Tu, on the surface of human pathogens *S. aureus* and *Mycoplasma pneumoniae*, and the porcine pathogen *M. hyopneumoniae*. Protein cleavage events were mapped using a systems wide dimethyl labelling protocol that allows for the identification of modified N-terminal peptides (neo-N-termini) by liquid chromatography tandem mass spectrometry (LC-MS/MS) and enabled us to determine how Ef-Tu is processed and presented on the cell surfaces of these pathogens. We further characterised the non-canonical functions of Ef-Tu from *M. pneumoniae* (Mpn_Ef-Tu_) and show that it is a multifunctional protein that can not only bind to and activate plasminogen in the presence of host activators, but is also capable of binding to structurally and chemically diverse host molecules.

## Results

### Bioinformatic analysis of Mhp_Ef-Tu_, Sa_Ef-Tu_ and Mpn_Ef-Tu_

The amino acid sequences of Ef-Tu from *M. pneumoniae* (Mpn_Ef-Tu_), *M. hyopneumoniae* (Mhp_Ef-Tu_), and *S. aureus* (Sa_Ef-Tu_) share 60.7% sequence identity. Mpn_Ef-Tu_ resides on the cell surface of *M. pneumoniae* and binds fibronectin^2^. The fibronectin-binding regions have been mapped and are located at the end of domain I and at the beginning of domain II^89, 90^ and most of domain III is also involved in binding fibronectin^89^. It is not known if sequence conservation in fibronectin-binding regions of Mhp_Ef-Tu_ and Sa_Ef-Tu_ is sufficient to afford these Ef-Tu homologs the ability to bind fibronectin. Several Mycoplasma species^73, 91^ and *S. aureus* ^92–94^ are known to interact with heparin. Putative heparin-binding domains were computationally predicted and mapped onto each of the Ef-Tu molecules (Fig. 1). Several of these were conserved in all three Ef-Tu sequences in domains I, II and III.

### Mhp_Ef-Tu_, Sa_Ef-Tu_ and Mpn_Ef-Tu_ are accessible on the bacterial surface and are retained during heparin-agarose chromatography

**Figure 2:**
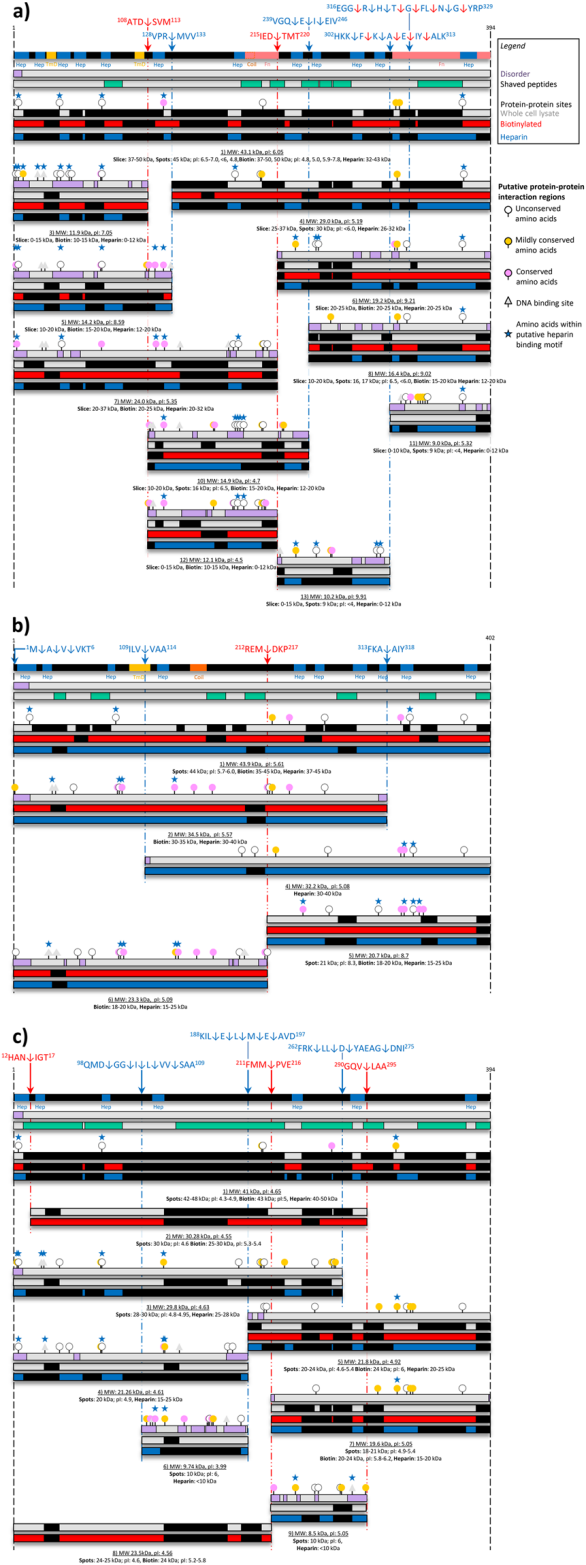
Binding to heparin by Ef-Tu and cleavage fragments of Ef-Tu. Panel a–c show Mpn_Ef-Tu_, Mhp_Ef-Tu_, and Sa_Ef-Tu_, and surface accessible cleavage fragments of these molecules retained during heparin-agarose chromatography respectively. Peptides identified (black boxes within coloured bars) by mass spectrometry within Ef-Tu fragments were obtained from 1D and 2D SDS-PAGE of bacterial whole cell lysates (grey bars), avidin affinity chromatography of biotinylated surface proteins (red bars), and heparin-agarose affinity chromatography (blue bars). Full length Ef-Tu molecules are represented as black bars. Cleavage products of Ef-Tu are also shown. Cleavage sites were identified by identifying dimethyl labelled peptides (blue arrows and broken lines) and by characterising semi-tryptic peptides generated after trypsin digestion (red arrows and broken lines). Exact cleavage sites are shown in the amino acid sequences above the black bar. For *M. pneumoniae*, the two fibronectin-binding regions (salmon boxes, Fn) and two putative transmembrane domains described by^89, 90^ were included. Bioinformatic tools such as ScanProsite^144^, TMpred^142^, COILS^143^ and Meta-Disorder^145^ were used to predict putative heparin-binding motifs (Hep, blue boxes), transmembrane domains (TmD, yellow box for *M. hyopneumoniae*), coiled-coils (Coil, orange boxes), and disordered regions (purple boxes in grey bar), respectively. Peptides released from trypsin shaving of cells are shown as the green boxes in the grey bar. Circles just above fragments denote amino acid positions that are predicted to be surface exposed and represent putative protein-protein interaction regions (visual cues can be seen on the right of the cleavage map, sites listed in Tables S2A, S2B, and S2C). Those marked with an additional star denote amino acid residues that fall within predicted putative heparin-binding domains. White circles mark evolutionary unconserved binding regions, whilst yellow circles are mildly conserved and pink are highly conserved. Amino acid positions marked by grey triangles depict predicted nucleic acid interaction regions.

LC-MS/MS analysis of tryptic peptides released from the cell surface of *S. aureus*, *M. pneumoniae* and *M. hyopneumoniae* were separately mapped to Sa_Ef-Tu_, Mpn_Ef-Tu_ and Mhp_Ef-Tu_ respectively_._ In other experiments, tryptic peptides generated by digesting biotinylated cell surface proteins that were captured by avidin agarose chromatography were also separately mapped to Sa_Ef-Tu_, Mpn_Ef-Tu_ and Mhp_Ef-Tu_. Peptides identified by mass spectrometry from both techniques spanned the entire length of Ef-Tu, (Figure S1) consistent with the hypothesis that a sub-population of Ef-Tu molecules are exposed on the cell surface of the three pathogens (Fig. 2) while the remainder perform an essential function in the cytosol. Tryptic peptides spanning the length of Sa_Ef-Tu_, Mpn_Ef-Tu_ and Mhp_Ef-Tu_ were also characterised when LC-MS/MS analysis was performed on tryptic digests of high salt (>500 mM) eluents of proteins that were retained on heparin agarose (Fig. 2).

### Mhp_Ef-Tu_, Sa_Ef-Tu_ and Mpn_Ef-Tu_ are cleaved on the bacterial cell surface

As part of a larger study that sought to identify the repertoire of proteins in *M. pneumoniae*, *M. hyopneumoniae* and *S. aureus* that are targets of proteolytic processing events, we employed a dimethyl labelling protocol to tag N-terminal peptides and identify precise endoproteolytic cleavage sites (Table 1). Further evidence that Sa_Ef-Tu_, Mpn_Ef-Tu_ and Mhp_Ef-Tu_ are targets of protein cleavage events was obtained by LC-MS/MS analysis of i) SDS-PAGE gel slices separately loaded with biotinylated *M. pneumoniae*, *M. hyopneumoniae* and *S. aureus* surface proteins captured by avidin chromatography, ii) bacterial proteins that eluted from heparin agarose using high salt (>500 mM NaCl), iii) protein spots representing bacterial whole cell lysates and surface biotinylated proteins separated by 2D-PAGE, and iv) size fractioned whole cell lysate proteins resolved by SDS-PAGE.

**Table 1:** Dimethyl labelled and semi-tryptic peptides identified in Mpn_Ef-Tu_, Mhp_Ef-Tu_ and Sa_Ef-Tu_.

No.	ID	Peptide Sequence	Score	E-value
	**Dimethyl Labelled peptides from Mpn**_**Ef-Tu**_			
1	N1	K.^58^ **A**RGITINSAHVEYSSDKR^75^.H	41	3.00E^−03^
	N2	R.^60^ **G**ITINSAHVEYSSDKR^75^.H	94	1.20E^−08^
3	N3	R.^131^ **M**VVFLNK^137^.C	57	1.30E^−03^
5	N4	Q.^242^ **E**IEIVGLRPIR^252^.K	48	3.30E^−03^
	N5	E.^243^ **I**EIVGLRPIR^252^.K	35	0.033
	N6	I.^244^ **E**IVGLRPIR^252^.K	37	0.014
7	N7	K.^305^ **F**KAEIYALKKEEGGR^319^.H	110	9.40E^−09^
	N8	K.^307^ **A**EIYALKKEEGGR^319^.H	69	2.60E^−05^
8	N9	R.^320^ **H**TGFLNGYRPQFYFR^334^.T	61	2.80E^−05^
	N10	H.^321^ **T**GFLNGYRPQFYFR^334^.T	39	2.90E^−03^
	N11	N.^326^ **G**YRPQFYFR^334^.T	43	1.70E^−03^
	**Semi-tryptic C-terminal Peptides from Mpn**_**Ef-Tu**_			
6	S1	R.^253^KAVVTGIEMFKKEL**D** ^267^.S	47	4.50E^−04^
	S2	R.^253^KAVVTGIEMFKKELD**S** ^268^.A	56	4.50E^−05^
	S3	R.^253^KAVVTGIEMFKKELDSAM**A** ^273^.G	55	7.30E^−05^
	S4	R.^253^KAVVTGIEMFKKELDSAMA**G** ^272^.D	95	4.50E^−09^
	S5	R.^253^KAVVTGIEMFKKELDSAMAGDN**A** ^275^.G	96	3.30E^−09^
	S6	R.^253^KAVVTGIEMFKKELDSAMAGDNA**G** ^276^.V	113	2.90E^−09^
	S7	R.^253^KAVVTGIEMFKKELDSAMAGDNAGVL**L** ^279^.R	55	2.00E^−05^
7	S8	R.^290^GQVLAKPGSIKPHKK**F** ^305^.K	46	1.50E^−04^
	S9	R.^290^GQVLAKPGSIKPHKKFK**A** ^307^.E	61	2.10E^−06^
	S10	R.^290^GQVLAKPGSIKPHKKFKAE ^308^.I	27*	4.40E^−03^
	S11	R.^290^GQVLAKPGSIKPHKKFKAEI**Y** ^310^.A	38	2.40E^−03^
8	S12	R.^290^GQVLAKPGSIKPHKKFKAEIYALKKEEG**G** ^318^.R	85	7.00E^−08^
	S13	R.^290^GQVLAKPGSIKPHKKFKAEIYALKKEEGGR**H** ^320^.T	24	8.20E^−03^
	S14	R.^290^GQVLAKPGSIKPHKKFKAEIYALKKEEGGRH**T** ^321^.G	15*	0.048
	S15	R.^290^GQVLAKPGSIKPHKKFKAEIYALKKEEGGRHT**G** ^322^.F	48	3.00E^−04^
	S16	R.^290^GQVLAKPGSIKPHKKFKAEIYALKKEEGGRHTGFL**N** ^325^.G	25*	9.50E^−03^
	S17	R.^320^HTGFLN**G** ^326^.Y	21*	0.058
	**Semi-tryptic N-terminal Peptides from Mpn**_**Ef-Tu**_			
1	S18	I.^62^ **T**INSAHVEYSSDKR^75^.H	37	4.60E^−03^
2	S19	D.^111^ **S**VMPQTREHILLAR^124^.Q	65	7.00E^−05^
4	S20	D.^218^ **T**MTITGR^224^.G	41	0.041
6	S21	L.^267^ **D**SAMAGDNAGVLLR^280^.G	73	2.40E^−06^
	S22	D.^268^ **S**AMAGDNAGVLLR^280^.G	85	3.60E^−06^
	S23	S.^269^ **A**MAGDNAGVLLR^280^.G	58	1.30E^−03^
	S24	A.^270^ **M**AGDNAGVLLR^280^.G	53	7.20E^−04^
	S25	M.^271^ **A**GDNAGVLLR^280^.G	75	1.90E^−05^
	S26	A.^272^ **G**DNAGVLLR^280^.G	59	7.70E^−04^
	S27	G.^273^ **D**NAGVLLR^280^.G	57	1.30E^−03^
	S28	D.^274^ **N**AGVLLR^280^.G	42	0.031
8	S29	H.^321^ **T**GFLNGYRPQFYFR^334^.T	76	6.00E^−06^
	S30	T.^322^ **G**FLNGYRPQFYFR^334^.T	47	3.00E^−03^
	S31	G.^323^ **F**LNGYRPQFYFR^334^.T	77	3.00E^−05^
	S32	L.^325^ **N**GYRPQFYFR^334^.T	62	5.30E^−05^
	S33	N.^326^ **G**YRPQFYFR^334^.T	47	2.90E^−04^
	S34	G.^327^ **Y**RPQFYFR^334^.T	59	1.40E^−03^
9	S35	C.^370^ **E**KGSKFSIR^378^.E	66	1.30E^−03^
	S36	C.^370^ **E**KGSKFSIREGGR^382^.T	35	6.60E^−03^
	**Dimethyl Labelled peptides from Mhp**_**Ef-Tu**_			
1	N1	M.^2^ **A**VVKTTGKKDFR^14^.S	84	5.70E^−07^
	N2	A.^3^ **V**VKTTGKKDFR^14^.S	36	0.021
	N3	V.^4^ **V**KTTGKKDFR^14^.S	34	0.019
2	N4	V.^112^ **V**AATDGPMPQTR^123^.E	74	1.70E^−05^
4	N5	A.^316^ **A**IYALKKEEGGR^327^.H	50.1	3.00E^−05^
	**Semi-tryptic N-terminal Peptides from Mhp**_**Ef-Tu**_			
3	S1	M.^215^ **D**KPFLMAVEDVFTITGR^231^.G	68	2.50E^−05^
	**Dimethyl Labelled peptides from Sa**_**Ef-Tu**_			
2	N1	D.^101^ **G**GILVVSAADGPMPQTR^117^.E	98	2.90E^−05^
	N2	G.^103^ **I**LVVSAADGPMPQTR^117^.E	81	1.30E^−03^
	N3	I.^104^ **L**VVSAADGPMPQTR^117^.E	104	5.90E^−06^
	N4	L.^105^ **V**VSAADGPMPQTR^117^.E	91	1.10E^−04^
	N5	V.^107^ **S**AADGPMPQTR^117^.E	69	9.90E^−03^
3	N6	N.^137^ **K**VDMVDDEELLELVEMEVR^155^.D	80	3.10E^−03^
	N7	D.^140^ **M**VDDEELLELVEMEVR^155^.D	78	3.40E^−03^
4	N8	L.^191^ **E**LMEAVDTYIPTPER^205^.D	78	3.60E^−03^
	N9	E.^192^ **L**MEAVDTYIPTPER^205^.D	78	3.20E^−03^
	N10	L.^193^ **M**EAVDTYIPTPER^205^.D	72	2.80E^−03^
	N11	M.^194^ **E**AVDTYIPTPER^205^.D	103	8.40E^−06^
	N12	E.^195^ **A**VDTYIPTPER^205^.D	77	9.60E^−04^
7	N13	K.^265^ **L**LDYAEAGDNIGALLR^280^.G	98	4.10E^−05^
	N14	L.^267^ **D**YAEAGDNIGALLR^280^.G	88	1.80E^−04^
	N15	D.^268^ **Y**AEAGDNIGALLR^280^.G	89	2.40E^−04^
	N16	G.^273^ **D**NIGALLR^280^.G	67	1.80E^−02^
9	N17	R.^335^ **T**TDVTGVVHLPEGTEMVMPGDNVEMTVELIAPIAIEDGTR^374^.F	86	7.70E^−07^
	**Semi-tryptic N-terminal Peptides from Sa**_**Ef-Tu**_			
8	S1	V.^293^ **L**AAPGSITPHTEFK^306^.A	106	3.20E^−05^
5	S2	M.^214^ **P**VEDVFSITGR^224^.G	80	0.010
1	S3	N.^15^ **I**GTIGHVDHGK^28^.T	80	1.10E^−04^
6	S4	F.^263^ **R**KLLDYAEAGDNIGALLR^280^.G	91	1.20E^−03^

Identified peptides have a Mascot score >33 and an E-value <0.05 unless marked with a *. Peptides marked with a *implies the peptide score was <33 but still lies within major cleavage site. The exact site of cleavage is to the left of the amino acid that is bold and underlined for N-terminal cleavage fragments and to the right of C-terminal cleavage fragments. Amino acid numbers are written at the start and end of each peptide identified by LC-MS/MS.

Of the 15 cleavage fragments of Mpn_Ef-Tu_ identified in this study, 11 were identified in the biotinylated 1D and 2D SDS-PAGE. Notably, three of the four cleavage fragments derived from Mhp_Ef-Tu_ and two of six fragments of Sa_Ef-Tu_ that were enriched during heparin affinity chromatography were also identified in biotinylation experiments (Figures S4, S5 and S6). Ten, four and six cleavage fragments that span different regions of Mpn_Ef-Tu_, Mhp_Ef-Tu_ and Sa_Ef-Tu_ respectively were recovered from a heparin agarose chromatography using salt concentrations well above the physiological concentration of 150 mM. All the fragments recovered from heparin affinity chromatography across all three pathogens contained at least one of the predicted heparin-binding domains that reside within Mpn_Ef-Tu_, Mhp_Ef-Tu_ and Sa_Ef-Tu_. These data suggest that the processing events that generate Ef-Tu cleavage fragments, occur on the surface of each of these pathogens and that the fragments may retain an ability to interact with high sulfated glycosaminoglycans such as heparin. To ascertain the nature of the protease(s) responsible for Ef-Tu surface cleavage, the MEROPs database was used to search 56 cleavage events. However, no strong predictions could be made after searching both P4-P3-P2-P1↓P1′-P2′-P3′-P4′ and P2-P1↓P1′-P2′ cleavage motifs.

### Processing events expose new predicted surface macromolecule interaction sites

A single heparin-binding consensus motif (XBBBXXBX, where B is a basic residue) with the sequence DKRHYAHV is found within the amino acid sequences of Sa_Ef-Tu_, Mpn_Ef-Tu_, and Mhp_Ef-Tu_, yet we found several Ef-Tu fragments that were retained during heparin agarose chromatography that did not span this motif. Sa_Ef-Tu_, Mpn_Ef-Tu_, and Mhp_Ef-Tu_ sequences were examined for additional motifs enriched with clustered basic residues. In Mpn_Ef-Tu_ we identified 12 putative heparin-binding motifs dispersed throughout the protein (Table S1). Many of these putative heparin-binding motifs, particularly sequences ^37^aKegKsaatRy^47^, ^183^pKweaKiHd^191^ and ^248^lRpiRKa^254^ were localised to non-essential regions defined here as evolutionary unconserved regions (See S9 - Supplementary Materials: Bioinformatics and Table S1). Using ISIS^95^, which predicts protein-protein interaction (PPI) sites from sequence information, Mpn_Ef-Tu_ is predicted to have eight surface exposed PPI sites that are capable of binding macromolecules (Table S2A) such as glycosaminoglycans including four that reside within putative heparin-binding motifs ^2^a**Re**KfdRsKpHv^13^, ^73^ **d**KRHyaHv^80^ and ^370^e**K**gsKfsiReggRt^383^ (Table S1). Notably, the key residues (underlined and in bold) in the four binding sites were all unconserved residues as determined by ConSurf^96^. Putative heparin-binding fragments derived from Mpn_Ef-Tu_ typically displayed more putative PPI sites and were more intrinsically disordered than the parent molecule and some fragments displayed putative nucleic acid interaction sites (Fig. 2), which are absent in the unprocessed, parent molecule. Additionally, three short linear motifs located in unconserved regions of Ef-Tu that were not predicted binding sites in the parent molecule were predicted to be exposed in Mpn_Ef-Tu_ fragment 3 (^37^ **aKegKs**aat**Ry** ^47^), fragment 4 (^183^ **p**KweaKiHd^191^), fragment 5 (^37^ **aKegK**saatRy^47^), fragment 6 (^248^lR**p**i**R**Ka^254^), fragment 7 (^37^aK**e**gKsaatRy^47^ and ^183^ **pKwea**KiHd^191^), fragment 10 (^183^ **p**K**w**e**a**Ki**H**d^191^), fragment 12 (^183^ **pKweaK**i**H**d^191^) and fragment 13 (^248^ **lRpiR**Ka^254^) (Table S1).

### Molecular modelling of Ef-Tu

The prediction tool MODELLER^97^ was used to predict the structures of Ef-Tu for all three pathogens based on Ef-Tu from *E. coli*. For the *M. pneumoniae* prediction, the *E. coli* Ef-Tu (PDB: 4G5G_A) had a structure ID percentage of 70.5% and a zDOPE score of −0.93. For *M. hyopneumoniae*, the *E. coli* Ef-Tu (PDB: 1DG1_H) had a structure ID percentage of 68.6% and a zDOPE score of −0.72. For *S. aureus*, the *E. coli* Ef-Tu (PDB: 1DG1_H) had a structure ID percentage of 75.1% and a zDOPE score of −0.88. All nine distinct cleavage sites for *M. pneumoniae* and *S. aureus* and four sites for *M. hyopneumoniae* have all been mapped in the ribbon structures (Figure S2). Cleavage sites located in regions that are predicted to release the three domains are mostly surface accessible within the molecule. The location and accessibility of the heparin-binding domains in Mpn_Ef-Tu_, Mhp_Ef-Tu_ and Sa_Ef-Tu_ and the two published fibronectin-binding domains in Mpn_Ef-Tu_ are depicted in Figure S3.

### Mpn_Ef-Tu_ and Mhp_Ef-Tu_ are potential multifunctional binding proteins

**Figure 3:**
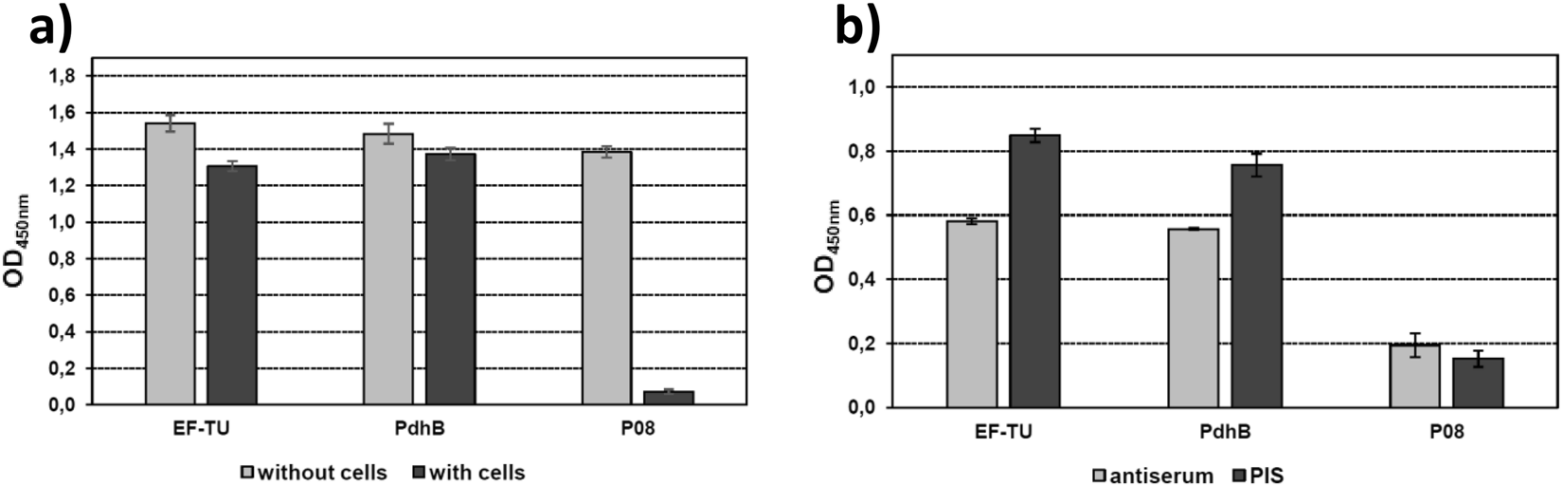
Binding of rMpn_Ef-Tu_ to human A549 epithelial cells. (**a**) A549 cells (‘with cells’) were bound to wells of a 96-well mictrotitre plate and incubated with rEf-Tu. Bound rMpn_Ef-Tu_ was detected with antisera raised against rMpn_Ef-Tu_. rPdhB and rP08 were used as a positive and negative control^98^, respectively. Bars represent standard deviation of eight replicates. (**b**) rMpn_Ef-Tu_ was incubated with either antisera raised against rMpn_Ef-Tu_ or pre-immune sera (PIS) and added to A549 cells in ELISA plates. rPdhB and rP08 and the corresponding antisera were used as a positive and negative control^98^, respectively. Bars represent standard deviation of eight replicates.

**Figure 4:**
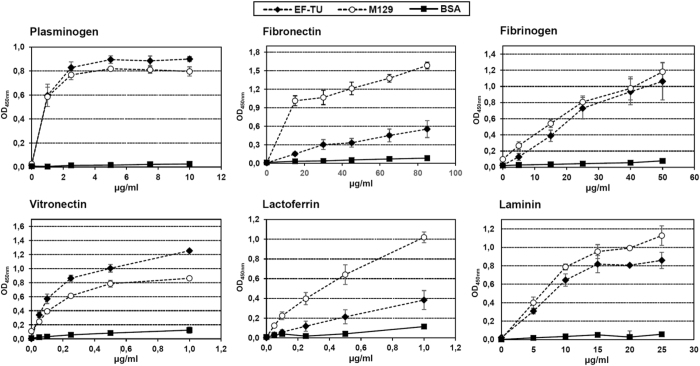
Microtitre plate binding assays depicting the interaction of rMpn_Ef-Tu_ with human proteins. Wells of microtitre plates were coated with rMpn_Ef-Tu_ and incubated with increasing concentrations of host proteins. Antisera against each of the host proteins was used to detect interaction with rMpn_Ef-Tu_. *M. pneumoniae* cells and BSA were used as a positive and negative control, respectively. Bars represent standard deviation of eight replicates.

It was notable that Mpn_Ef-Tu_ was recovered from *M. pneumoniae* native cell lysates that were loaded onto affinity columns coupled with A549 epithelial cell surface proteins, fetuin, fibronectin, actin or plasminogen (Figure S4). Consistent with these data, rMpn_Ef-Tu_ bound to immobilized A594 cells in microtitre plate binding assays (Fig. 3a). Proteins that bind (recombinant pyruvate dehydrogenase subunit B) and that do not bind (P08 fragment of P1 adhesin) to A594 cells were used positive and negative controls respectively^98^. Binding of rMpn_Ef-Tu_ to A594 cells was partially inhibited when anti-rMpn_Ef-Tu_ antibodies, but not pre-immune antiserum, was present (Fig. 3b). Mhp_Ef-Tu_ was recovered from native cell lysates of *M. hyopneumoniae* that were loaded onto affinity columns coupled with PK15 epithelial cell surface proteins, fibronectin, actin, or plasminogen (Figure S4). Mpn_Ef-Tu_ has previously been shown to bind fibronectin^2^ and we independently confirmed this in microtitre plate binding assays. Furthermore, our binding assay suggests that *M. pneumoniae* encodes fibronectin-binding proteins other than Ef-Tu (Fig. 4). Mpn_Ef-Tu_, and nine of the fifteen cleavage fragments of Mpn_Ef-Tu_, were recovered from affinity columns loaded with fibronectin (Figure S3). Of the nine cleavage fragments, seven spanned the known fibronectin-binding regions described previously (see Fig. 1)^89, 90^. We also identified fragments from columns coupled to fibronectin that spanned the N-terminus of Mpn_Ef-Tu_ suggesting that other fibronectin-binding domains are yet to be identified in this molecule. Mhp_Ef-Tu_ and six cleavage fragments of Mhp_Ef-Tu_ were retained by columns coupled with fibronectin (Figure S5). The cleavage fragments spanned the N- and C-terminal ends, as well as the central region of Mhp_Ef-Tu_ suggesting that it may contain fibronectin-binding domains.

Ten fragments spanning different regions of Mpn_Ef-Tu_ (Figure S4) and one Mhp_Ef-Tu_ fragment (Figure S5) were identified from affinity columns coupled with biotinylated surface proteins derived from A549 and PK-15 cells, respectively. Mpn_Ef-Tu_ and Mhp_Ef-Tu_, and fragments derived from them, were recovered from actin-coupled columns (Figures S4 and S5). Five fragments of Mpn_Ef-Tu_ were recovered during affinity chromatography using fetuin as bait (Figure S4).

*M. pneumoniae* ^99–101^ and *M. hyopneumoniae* ^78, 81^ have both been shown to bind plasminogen onto their cell surface and assist with its conversion to plasmin. In the current study, Mpn_Ef-Tu_ and Mhp_Ef-Tu_ were both recovered during plasminogen agarose chromatography. Fragments spanning different regions of Mpn_Ef-Tu_ (Figure S4) and Mhp_Ef-Tu_ (Figure S5) were recovered from plasminogen coupled agarose beads.

### Mpn_Ef-Tu_ is a multifunctional adhesin

**Figure 5:**
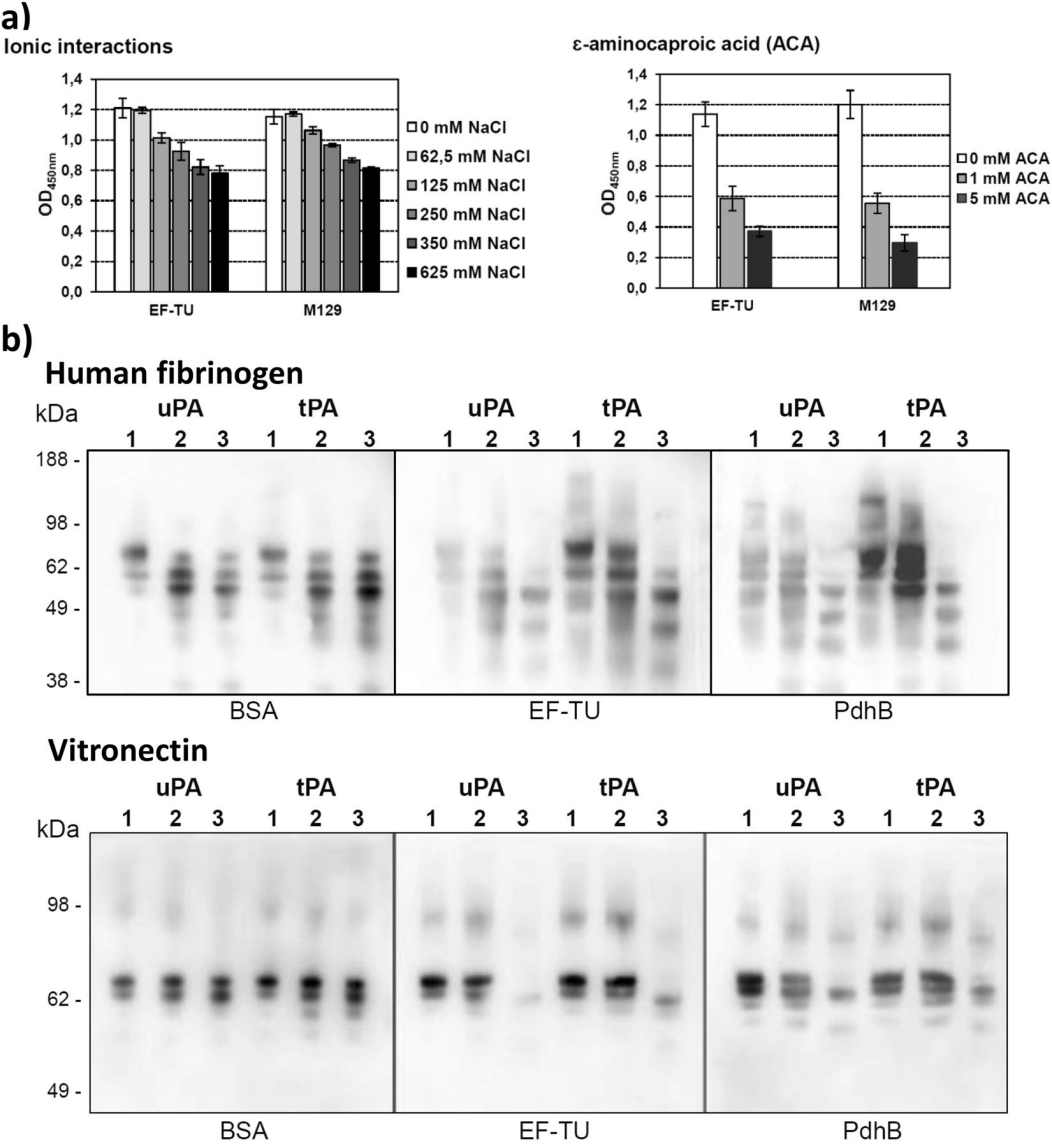
Influence of ions and lysine analog ACA on binding of rMpn_Ef-Tu_ to plasminogen and degradation of human fibrinogen and vitronectin by activated plasminogen. (**a**) Microtitre plate wells were coated with rMpn_Ef-Tu_ and incubated with plasminogen and increasing concentrations of either NaCl (‘ionic interactions’) or ε-aminocaproic acid (‘ACA’). Bound plasminogen was detected with anti-plasminogen antibodies. In control experiments *M. pneumoniae* cells were coated onto microtitre plates and incubated with plasminogen and increasing concentrations of either NaCl or ACA. Bars represent standard deviation of eight replicates. (**b**) Fibrinogen or vitronectin was mixed with either urinary plasminogen activator (uPA) or tissue plasminogen activator (tPA) and added to microtitre plates previously coated with rMpn_Ef-Tu_ and plasminogen. Samples were separated by SDS-PAGE, blotted onto nitrocellulose membrane and probed with anti-vitronectin and anti-fibrinogen antisera. Lane 1 is at 0 hours, lane 2 is after over-night incubation without plasminogen, and lane 3 is after over-night incubation with plasminogen. Full length blots can be seen in Figure S9.

Antibodies raised against rMpn_Ef-Tu_ were used to show that Mpn_Ef-Tu_ resides on the surface of colonies of *M. pneumoniae* (Figure S7). Our surfaceome studies (unpublished data) identified candidate proteins that could be used as a negative control for these studies and antibodies raised against recombinant 1-phosphofructokinase (FruK) from *M. pneumoniae* were used for this purpose (Figure S7). To further investigate the binding capabilities of rMpn_Ef-Tu_, we examined the ability of the molecule to interact with a range of host molecules. rMpn_Ef-Tu_ bound to fetuin (K_D_ = 53 ± 14 nM), actin (K_D_ = 19 ± 3 nM) and heparin (K_D_ = 42.5 ± 1.5 nM) in the nanomolar range and to plasminogen (K_D_ = 933 ± 388 nM) in the micromolar range, using microscale thermophoresis (Figure S8). We extended these studies using microtitre plate binding assays to confirm that rMpn_Ef-Tu_ binds plasminogen and fibronectin, and also show that rMpn_Ef-Tu_ binds fibrinogen, vitronectin, lactoferrin and laminin in a dose dependent manner (Fig. 4). Binding of rMpn_Ef-Tu_ to plasminogen was significantly reduced by the addition of an increasing concentration of NaCl and ε-aminocaproic acid (Fig. 5a). Notably, ε-aminocaproic acid was effective at blocking interactions between *M. pneumoniae* and plasminogen while high concentrations of NaCl were less effective (Fig. 5a). These data suggest that lysine residues play a significant role in binding interactions between Ef-Tu and plasminogen, and *M. pneumoniae* cells and plasminogen.

In the presence of plasminogen activators tPA and uPA plasminogen bound to rMpn_Ef-Tu_ is converted to plasmin and can degrade fibrinogen and vitronectin (Fig. 5b). Collectively these studies highlight the widespread multifunctional capabilities of Ef-Tu and the cleavage fragments derived from it.

## Discussion

Ef-Tu moonlights on the cell surface of *S. aureus*, *M. pneumoniae* and *M. hyopneumoniae*, three phylogenetically diverse, pathogenic bacteria that belong to the low G + C Firmicutes. Using a combination of microscale thermophoresis and microtitre plate binding assays we show that rMpn_Ef-Tu_ binds strongly to heparin (K_D_ = 42.5 ± 1.5 nM), fetuin (K_D_ = 53 ± 14 nM) and actin (K_D_ = 19 ± 3 nM), as well as to laminin, plasminogen, vitronectin, lactoferrin, fibronectin, and fibrinogen. Plasminogen bound to rMpn_Ef-Tu_ can be converted to plasmin in the presence of plasminogen activators tPA and uPA (Fig. 5). We also extend these finding by showing that Sa_Ef-Tu_, Mpn_Ef-Tu_ and Mhp_Ef-Tu_ are targets of processing events on the cell surface of these bacterial pathogens but the biological significance of this warrants further investigation (see below). Molecules are not strictly confined to compartments in the bacterial cell and can perform novel functions at different cellular locations^24, 25, 54, 102–105^. Much remains to be learnt about how proteins, especially those lacking signal motifs, localise on bacterial cell surfaces.

We sought to gain a better understanding of how Ef-Tu has evolved to be a multifunctional binding protein. Sa_Ef-Tu_, Mpn_Ef-Tu_ and Mhp_Ef-Tu_ all putatively bind heparin, each sharing the consensus heparin-binding motif XBBBXXBX (sequence: dKRHyaHv) as well as a number of other heparin-binding motifs (see Fig. 1 and Table S1). It is notable that while this motif (dKRHyaHv) is conserved in the Ef-Tu from *M. pneumoniae*, *M. hyopneumoniae* and *S. aureus* only part of the motif, with the sequence RHyaHv, is conserved in Ef-Tu from other bacterial sources. The addition of DK residues is predicted to impart a putative PPI site. Twelve putative heparin-binding motifs identified in Mpn_Ef-Tu_ (Table S1) were predicted to predominantly localise to non-essential, unconserved regions of the molecule that do not unduly influence its ability to function as an elongation factor. Short linear motifs (SLiMs) typically ranging from three to ten amino acids play crucial roles in mediating PPIs^106–108^. In eukaryotes, these motifs are typically located in intrinsically unstructured, disordered regions of proteins that impart plasticity and are reported to favour transient, low affinity and reversible interactions^106, 109^. Notably, Mpn_Ef-Tu_ formed strong interactions with fetuin, heparin, and actin suggesting that the accumulation of SLiMs may be sufficient to form high affinity interactions.

Positively charged amino acids in SLiMs play a crucial role in interactions between proteins and highly sulphated glycosaminoglycans such as heparin^110^, and other molecules such as actin^111^, plasminogen^112^, DNA^113, 114^ and fibronectin^69, 84, 115^. Here we identified SLiMs enriched in positively charged amino acids in different regions of Mpn_Ef-Tu_, including sequences ^37^aKegKsaatRy^47^, ^183^pKweaKiHd^191^, and ^248^lRpiRKa^254^, and identified eight surface exposed PPI sites, including three that reside within putative heparin-binding motifs ^2^a**Re**KfdRsKpHv^13^, ^73^ **d**KRHyaHv^80^, and ^370^e**K**gsKfsiReggRt^383^. It is notable that the lysine analog, ε-amino caproic acid, was shown to be a potent inhibitor of interactions between Mpn_Ef-Tu_ and plasminogen, and M129 whole cells and plasminogen, underscoring the important role played by positively charged amino acids in binding interactions with host molecules (Fig. 5a). Overlapping SLiMs are frequently identified in multifunctional proteins^106, 116^. In *M. hyopneumoniae*, the C-terminal sequence ^1070^KKsslKvKitvK^1081^ in the multifunctional cilium adhesin, P97 binds both heparin and fibronectin^84^ and overlapping peptides from a region within phosphoglycerate kinase from group B streptococcus strain NCS13 with sequence ^203^sKvsdKigvienlleKadKv^222^ and ^213^enlleKadKvligggmtytf^232^ bind both actin and plasminogen^112^. Similarly, we were able to identify SLiMs enriched in positively charged amino acids in Sa_Ef-Tu_ and Mhp_Ef-Tu_. The accumulation of positively charged residues in SLiMs, possibly as a consequence of an A + T rich genome, facilitates binding to a wide range of host molecules in the low G + C Firmicutes. Our data is consistent with the proposition that the accumulation of surface exposed SLiMs represents a mechanism to generate protein multifunctionality in bacterial proteins.

*S. aureus* ^92^ and *M. hyopneumoniae* ^73–75, 82, 84, 87, 88^, display cell surface, heparin-binding proteins that are important to the pathogenic potential of these species. Interactions between heparin-binding proteins and target receptors in host cell membrane allow microbes to colonise a wide range of niche sites, traverse tissue barriers and disseminate from their initial point of contact and form biofilms^117^. *S. aureus* ^118, 119^, *M. pneumoniae* ^120, 121^ and *M. hyopneumoniae* (our unpublished data) are all capable of forming biofilms. The extracellular matrix of *S. aureus* biofilms is derived from a mixture of eDNA and cytoplasmic proteins^118, 122–127^ and electrostatic interactions between cytoplasmic proteins and eDNA is thought to tether cells together in *S. aureus* and mixed species biofilms^127^. In *S. aureus*, the addition of heparin increases biofilm production in a protein dependant manner which implies that heparin-binding proteins are important for biofilm development^92^. Notably, Ef-Tu has been identified on the surface of *S. aureus* under biofilm inducing conditions^122^. These observations lend weight to the hypothesis that the accumulation of positively charged amino acids in SLiMS represents a powerful mechanism to promote PPIs that underpin essential biological processes such as the formation and maintenance of biofilms.

Bacterial pathogens including *Campylobacter jejuni* ^69^, *Mycoplasma gallisepticum* ^86^, and *Chlamydia trachomatis* ^128^ process molecules that are secreted to the cell surface. In *M. hyopneumoniae*, processing of cilium adhesin families has been reported extensively and cleavage motifs have been mapped^73, 77, 80, 83^. Recently we showed that lactate dehydrogenase is cleaved on the surface of *M. hyopneumoniae* generating fragments with putative multifunctional binding capabilities^68^. In *M. pneumoniae*, cleavage fragments of the major adhesin P1 and DnaK have been shown to comprise part of the cytoskeletal attachment organelle complex^129^ and Mycoplasma derived lipoproteins are targets of processing events that release powerful immunomodulatory peptides^71, 130–132^. These observations prompted us to utilise a systems wide, protein dimethyl labelling strategy to investigate protein processing. Here we identified and characterised numerous processing sites in Ef-Tu derived from all three bacterial pathogens. Furthermore, our surface biotinylation studies indicate Mpn_Ef-Tu_, Mhp_Ef-Tu_ and Sa_Ef-Tu_, were a target of multiple processing events on the surfaces of *M. pneumoniae*, *M. hyopneumoniae* and *S. aureus*, respectively. Our work strongly suggests that the accumulation of positively charged residues in the SLiMs found in Ef-Tu facilitates binding to a wide range of host molecules, and potentially to eDNA and that protein cleavage events expand the functional complexity of proteins that moonlight on the cell surface. We propose that processing is a mechanism that has evolved to promote multifunctional behaviour more broadly and lends itself to the creation of novel binding sites in moonlighting proteins that retain a strict conformational structure needed to execute their canonical function.

Fifteen cleavage fragments of Mpn_Ef-Tu_ were identified in this study of which eleven reside on the cell surface. Unlike full length Mpn_Ef-Tu_, none of the fragments were retained in all six affinity chromatography columns, but five were identified in at least five affinity columns (fragments 5, 6, 7, 8, and 10 in Figure S4). Fragments 5, 8, and 10 were retained in columns coupled with: A549 surface proteins, fetuin, fibronectin, actin, and heparin. Fragments 6 and 7 were retained in columns coupled with: A549 surface proteins, fibronectin, actin, heparin, and plasminogen. Fragment 4 was identified in eluents from columns coupled with A549 surface proteins and heparin while Fragment 9 was identified in eluents from columns coupled with fetuin and actin (see Figure S4). These data indicate that retention of the fragments during affinity chromatography is dependent on the host molecule that is coupled to the agarose beads and the sequence of the Ef-Tu fragment. Further studies are needed to quantify the binding characteristics of fragments of Ef-Tu with host molecules.

Cleavage fragments of cytosolic proteins that moonlight on the cell surface add another layer of complexity to the concept of multifunctional proteins. We show that processing exposes SLiMs that would otherwise be inaccessible for interactions with potential binding partners. Recently, a peptidome study of a protease deficient strain of *Lactococcus lactis* identified 1800 distinct peptide fragments in spent growth medium that were derived from proteolytic activity targeting both surface accessible and cytosolically derived proteins^133^. Similar studies by the same group indicated that surface accessible proteins in other Firmicute species including *Listeria monocytogenes*, *Enterococcus faecalis* and *Streptococcus thermophilus* were also targeted by complex processing events^133^. Previously we have shown that processing events play an important role in the maturation of key adhesin families in pathogenic mycoplasma species^67, 72–86^. Here we extend these findings to show that surface proteolysis is critical in shaping the surface proteome more broadly and that processing represents a novel and under recognised mechanism to expand protein function.

In summary, Ef-Tu moonlights on the surface of bacteria where it is a target of proteolytic processing events. Computational analysis of fragments of Mpn_Ef-Tu_ suggest they are inherently more disordered and display putative PPI sites that are inaccessible in the parent molecule, generating unprecedented functional diversity on the cell surface. Further studies, using systems wide methodologies, are needed to determine how processing generates biologically important effector molecules and if protein processing is fundamental to the expansion of protein function in bacteria belonging to different phylogenetic clades.

## Experimental Section

A full description of the experimental section is listed in the S10 - Supplementary Materials 3.

### Strains and cultures and reagents

*M. pneumoniae* (M129 strain; ATCC 29342) was cultured in modified Hayflick’s medium at 37 °C in tissue culture flasks as described previously^134^.

*M. hyopneumoniae* (J strain) was cultured in modified Friis medium at 37 °C with shaking as described previously^135, 136^.

*S. aureus* (SH 1000 strain) was cultured in TSB (Oxoid, Hampshire, UK) at 37 °C with shaking and harvested during early stationary phase. Protease inhibitors (Roche Diagnostics^®^, North Ryde, Australia) in PBS were added to the cells during harvest and washes with PBS. For *S. aureus* lysis, cell pellets were freeze-dried overnight before added to pre-cooled metal milling canisters with 12 small metal beads. The canister was cooled in liquid nitrogen and milled at a maximum frequency of 30 Hz for 1 minute for 15 rounds; cooling in liquid nitrogen between rounds. Proteins were than solubilised in 7 M urea, 2 M thiourea, 50 mM LiCl, 50 mM Tris-HCl (pH 8.8), 1% (w/v) C7bZ0 with protease inhibitors followed by sonication at maximum intensity for 30 seconds for 20 rounds resting on ice in between.

Human lung carcinoma cells (A549; ATCC CCL-185) were cultured in RPMI 1640 medium (Invitrogen, Carlsbad, CA) supplemented with 10% heat inactivated fetal bovine serum at 37 °C with 5% CO_2_ in tissue culture flasks.

Porcine kidney epithelial (PK-15) cells were cultured in DMEM medium (Invitrogen) supplemented with 10% heat inactivated fetal bovine serum at 37 °C with 5% CO_2_ in tissue culture flasks.

Details about host proteins and human proteins used in this article are supplied in supplementary materials (S10.1).

### Enrichment of *M. pneumoniae*, *M. hyopneumoniae* and *S. aureus* surface proteins

#### Biotinylation

Biotinylation of the *M. pneumoniae* cell surface was carried out as described in^67^. *M. hyopneumoniae* and *S. aureus* cells were washed with PBS after centrifugation before the resuspending in EZ-link sulfo-NHS-biotin (Thermo Fisher Scientific, North Ryde, Australia). *M. hyopneumoniae* and *S. aureus* cells were biotinylated for 30 seconds and 1 minute, respectively. Quenching, lysis (for *M. hyopneumoniae*), avidin purification and western blotting were the same as for *M. pneumoniae*. Lysis for *S. aureus* cells is described above in section ‘Strains and cultures and reagents’.

### Triton X-114 phase extraction of biotinylated M. hyopneumoniae proteins

Triton X-114 phase extraction of proteins was carried out as described in^77, 83, 137^ and biotinylated surface proteins were purified by avidin column chromatography.

#### Trypsin shaving

Trypsin shaving of *M. pneumoniae* cells was carried out as described previously^75^ with modifications. Trypsin was added to adherent *M. pneumoniae* cells within tissue culture flasks, and *M. hyopneumoniae* and *S. aureus* cells were resuspended in trypsin.

### Preparation and separation of whole cell lysates for one- and two-dimensional gel electrophoresis

#### Whole cell lysis preparation

*M. pneumoniae* and *M. hyopneumoniae* whole cell lysates were prepared as previously described^75^. Lysis for *S. aureus* cells is described in section 'Strains and cultures and reagents' ﻿above. Proteins were reduced and alkylated with 5 mM tributylphosphine and 20 mM acrylamide monomers for 90 min at room temperature. Insoluble material was removed by centrifugation and five volumes of acetone added to precipitate protein. After centrifugation, the protein pellet was solubilized in 7 M urea, 2 M thiourea, 1% (w/v) C7BzO for one- and two-dimensional gel electrophoresis.

#### 1D and 2D SDS-PAGE protein separation

Protein separation was performed as described in^79, 82^. 80 μg of protein was separated for 1D SDS-PAGE and 250 μg of protein was cup-loaded for 2D SDS-PAGE separation.

#### Trypsin Digest

In-gel trypsin digestion was performed as described in^77^. After digestion, tryptic peptides were stored at 4 °C until needed for liquid chromatography tandem mass spectrometry.

### Heparin affinity chromatography

Affinity purification of heparin-binding proteins for *M. pneumoniae* was performed as described in^67^. *M. hyopneumoniae* cells were and lysed in 10 mM sodium phosphate, pH 7 with three 30 second rounds of sonication. *S. aureus* cells were lysed as described in section 1.1 except that protein was solubilised in 10 mM sodium phosphate, pH 7 with protease inhibitors followed by sonication at maximum intensity for 30 seconds for 4 rounds, resting on ice in between. After centrifugation, ~300 µg of soluble protein from both *M. hyopneumoniae* and *S. aureus* lysates were treated exactly the same as *M. pneumoniae*.

### Avidin purification of host-binding *M. pneumoniae* proteins

Purified fibronectin (Merck Millipore, Darmstadt, Germany), plasminogen (Merck Millipore), actin (Sigma, St. Louis, MO) and fetuin (Sigma) used in this section are described in supplementary section S10.1. Avidin purification of these host-binding *M. pneumoniae* proteins was carried out as described in^67^. Avidin purification of *M. pneumoniae* proteins that bind A549 surface proteins was performed as described in^67^.

### Avidin purification of host-binding *M. hyopneumoniae* proteins

Purified fibronectin (Merck Millipore), plasminogen (Sigma) and actin (Sigma) used in this section are described in supplementary section S10.1. Avidin purification of these host-binding *M. hyopneumoniae* proteins was performed as described in^84^. Avidin purification of *M. hyopneumoniae* proteins that bind PK-15 surface proteins was performed as described in^82^.

### Liquid chromatography tandem mass spectrometry (LC-MS/MS) and MS/MS data analysis

LC-MS/MS was performed as described in^82^. Mascot (Version 6.1) was used to search MS/MS data files as previously described^82^ with modifications (see supplementary section S10.2 for details).

### Expression and purification of rMpn_Ef-Tu_

Expression and purification of rMpn_Ef-Tu_ was performed in one of two methods as described by^100, 88^. Details and modifications to methods can be found in supplementary materials (section S10.3).

### Binding of rMpn_Ef-Tu_ to A549 cells

#### Binding assays

For this experiment and all subsequent experiments, animal experiments were approved by the ethical board of Landesdirektion Sachsen, Dresden, Germany (with the permit no. permit 24-9168.25-1/2011-1). ELISA experiments were carried out as described in^98^. Guinea pig rMpn_Ef-Tu_ antiserum (1:750) followed by anti-guinea pig IgG (1:1,000, Dako, Glostrup, Denmark) dilutions were used. Tetramethylbenzidine (Sigma) was added followed by 1 M HCl and absorbance was measured at 450 nm (620 nm as reference).

#### Influence of anti-rMpn_Ef-Tu_ on binding

Freshly grown A549 cells were used to coat wells in 96-well microtitre plates for 2 h at 37 °C as described in above ﻿in﻿ ‘Binding assays’. rMpn_Ef-Tu_ (10 µg/ml) was incubated with guinea pig rMpn_Ef-Tu_ antiserum or pre-immune serum (1:100) concentrations were used.

### Binding of rMpn_Ef-Tu_ to human proteins in ELISA

Purified human proteins used were supplied by Sigma and described in supplementary section S10.1. Binding of rMpn_Ef-Tu_ (15 µg/ml) to extracellular matrix proteins was performed as described previously^98^. The dilutions for the appropriate antisera are: (Sigma) anti-plasminogen: 1:2,500; anti-lactoferrin 1:5,000; anti-laminin 1:750; anti-vitronectin 1:5,000; anti-fibrinogen 1:3,000; anti-fibronectin 1:1,000. Followed by anti-rabbit IgG (Dako, Glostrup, Denmark) or anti-goat IgG (both 1:2,000).

### Microscale thermophoresis

Microscale thermophoresis to determine the binding affinities between Ef-Tu and a fluorescently labelled host protein was performed as described in^84^. Time for Microscale thermophoresis was set to 30 s with fluorescence set to 5 s before and 30 s after each run. Each sample was scanned with 40%, 60% and 80% MST Power. Dissociation curves were plotted with hot/cold, jump or thermophoresis settings to determine dissociation constant.

### Binding affinity of rMpn_Ef-Tu_ to plasminogen

#### Effect of NaCl on plasminogen-binding

Briefly, 96-well microtitre plates were coated with rMpn_Ef-Tu_ as described. Plasminogen (2.5 µg) together with increasing concentrations of NaCl were added to the wells and incubated for 1.5 h at 37 °C. Wells were incubated with rabbit anti-plasminogen (1:3,000) followed by anti-rabbit IgG (1:2,000). Detection was done as described above in ‘Binding assays’.

#### Effect of ε-aminocaproic acid on plasminogen-binding

ELISA was carried out as reported in^98^. In brief, the wells of ELISA plates were coated with rMpn_Ef-Tu_. 2.5 µg of plasminogen and increasing concentrations of ε-aminocaproic acid were added to the wells and incubated for 1.5 h at 37 °C. Wells were incubated with rabbit anti-plasminogen (1:3,000) followed by anti-rabbit IgG (1:2,000) and OD_420nm_ was measured.

### Plasminogen activation and degradation of human fibrinogen and vitronectin

Degradation of human fibrinogen and vitronectin by activated plasminogen was carried out as described in^98^. 10 µg/ml of human plasminogen was added to the wells which were then incubated with fibrinogen or vitronectin (each 15 µg/ml) and urinary plasminogen activator (uPA; Sigma) or tissue plasminogen activator (tPA; each 75 ng/ml; Sigma).

### Binding of anti- rMpn_Ef-Tu_ antibodies to *M. pneumoniae* whole cell lysate proteins

Freshly grown *M. pneumoniae* cells were harvested and used to coat wells in 96-well microtitre plate for 2 h at 37 °C as described previously^100^. Wells were blocked before adding guinea pig rMpn_Ef-Tu_ antisera (1:500) followed by anti-guinea pig IgG (1:1,000). As a control wells were incubated with guinea pig antisera raised against total *M. pneumoniae* proteins.

### Surface localisation of Ef-Tu on *M. pneumoniae*

#### Localisation of Ef-Tu on the surface of *M. pneumoniae* colonies

*M. pneumoniae* colonies were grown on PPLO agar plates and blotted onto nitrocellulose as described previously^100^. Antisera to PdhB and 1-phosphofructokinase (FruK) were used as positive and negative controls, respectively.

#### Surface localisation of Ef-Tu on *M. pneumoniae* cells

Immunofluorescence experiments were carried out as described in^100^. Again guinea pig antisera to PdhB and FruK were used as positive and negative controls, respectively.

### Dimethyl labelling and LC-MS/MS analysis of *M. pneumoniae*, *M. hyopneumoniae* and *S. aureus* proteins

#### Dimethyl labelling of proteins

Dimethyl labelling of proteins was performed as described previously^67, 68^.

#### LC-MS/MS of dimethyl labelled proteins

Dimethyl labelled proteins were analysed by two mass spectrometers; the Sciex 5600 and the Thermo Scientific Q Exactive™. For full technical set up and method details see supplementary materials (section S10.4).

### Bioinformatic analysis of Ef-Tu

Bioinformatic analysis of Ef-Tu used the online resources: ProtParam^138^, Clustal Omega^139^, SignalP 4.1 Server^140^, SecretomeP 2.0 Server^141^, TMpred^142^ and COILS (Addition of ‘yes’ to 2.5 fold weighting of positions a,d)^143^. The amino acid sequences of Mpn_Ef-Tu_ (Uniprot#: P23568), Mhp_Ef-Tu_ (Uniprot#: Q4A9G1) and Sa_Ef-Tu_ (Uniprot#: Q2G0N0) were analysed using a variety of bioinformatics tools. Conservation of amino acid positions in each protein were detected using The ConSurf server^96^. Putative heparin-binding sites were identified using the search patterns X-[HKR]-X(0,2)-[HKR]-X(0,2)-[HKR]-X and X-[HKR]-X(1,4)-[HKR]-X(1,4)-[HKR]-X via ScanProsite^144^. Putative protein-protein and protein-nucleic acid interaction sites were identified using ISIS^95^. Intrinsically disordered regions were predicted by Meta-Disorder^145, 146^, which combines the outputs from original prediction methods NORSnet, DISOPRED2, PROFbval and Ucon. Solvent accessibility of each amino acid position was ascertained using evolutionary information from multiple sequence alignments and a multi-level system^147^. Nucleotide, DNA and RNA binding regions were predicted by SomeNA^148^.

### Data availability statement

The datasets generated during and/or analysed during the current study are available from the corresponding author on reasonable request.

## Electronic supplementary material

Supplementary Information
