# Supplementary material for: Elongation factor Tu is a multifunctional and processed moonlighting protein
Source: Sci Rep. 2017 Sep 11;7:11227. doi: 10.1038/s41598-017-10644-z (PMC5593925; doi:10.1038/s41598-017-10644-z)
Supplement: Supplementary file 1 — Supplementary Information [file 41598_2017_10644_MOESM1_ESM.pdf]

Elongation factor Tu is a multifunctional and processed moonlighting protein

Michael Widjaja[1,†], Kate Louise Harvey[1,†], Lisa Hagemann[2,†], Iain James Berry[1], Veronica Maria Jarocki[1], Benjamin Bernard Armando Raymond[1], Jessica Leigh Tacchi[1], Anna Gründel[2], Joel Ricky Steele[1], Matthew Paul Padula[3], Ian George Charles[4], Roger Dumke[2¥], Steven Philip Djordjevic[1,3,¥*]

[1] The ithree institute, University of Technology Sydney, PO Box 123, Broadway, NSW, 2007, Australia.

[2] Technische Universität Dresden, Medizinische Fakultät Carl Gustav Carus, Institut für Medizinische Mikrobiologie und Hygiene, Fetscherstrasse 74, 01307 Dresden, Germany.

[3] Proteomics Core Facility, University of Technology Sydney, PO Box 123, Broadway, NSW, 2007, Australia.

[4] Institute for Food Research, Norwich Research Park, Norwich, NR4 7UA, UK.

† These authors contributed equally to this work

¥ These contributors share senior authorship

*Corresponding Author:

Prof. Steven P. Djordjevic

The ithree institute, University of Technology Sydney. PO Box 123, Broadway, NSW, 2007, Australia

Phone: +612 9514 4127

Fax: +612 9514 4143

Email: Steven.Djordjevic@uts.edu.au

Supplementary Tables and Figures

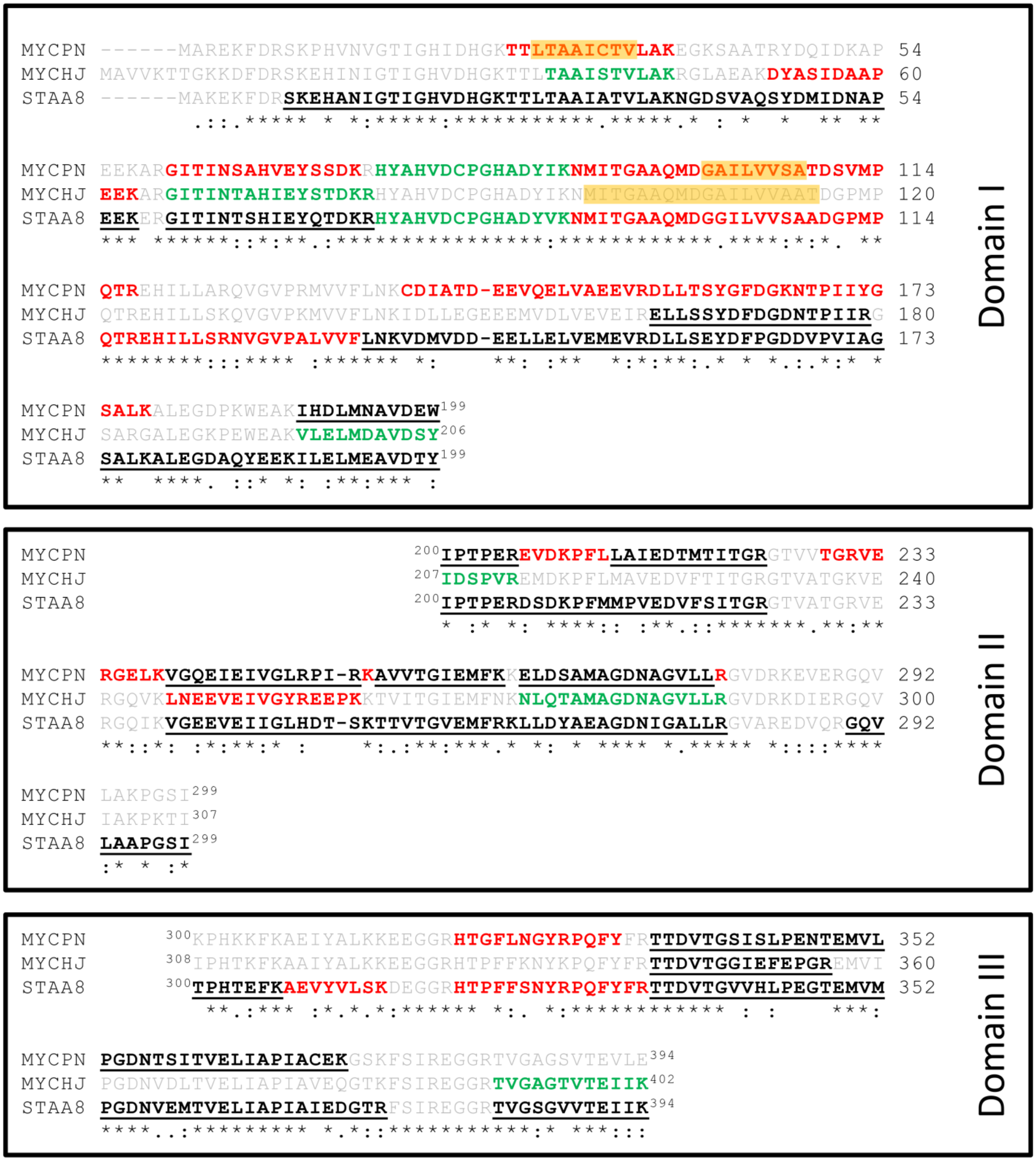

Figure S1. Peptides that map to Ef-Tu identified from surface biotinylation and shaving experiments. The sequence alignment for all three pathogens are shown separated into the three

domains of Ef-Tu. Peptides identified from surface trypsin shaving of whole cells are coloured green where red text indicate peptides from biotinylated surface proteins. Peptides that were identified from both experiments are highlighted as black underlined text. Yellow boxes indicate transmembrane domains for: *M. pneumoniae* adapted from [89] and *M. hyopneumoniae* predicted by TMpred [142].

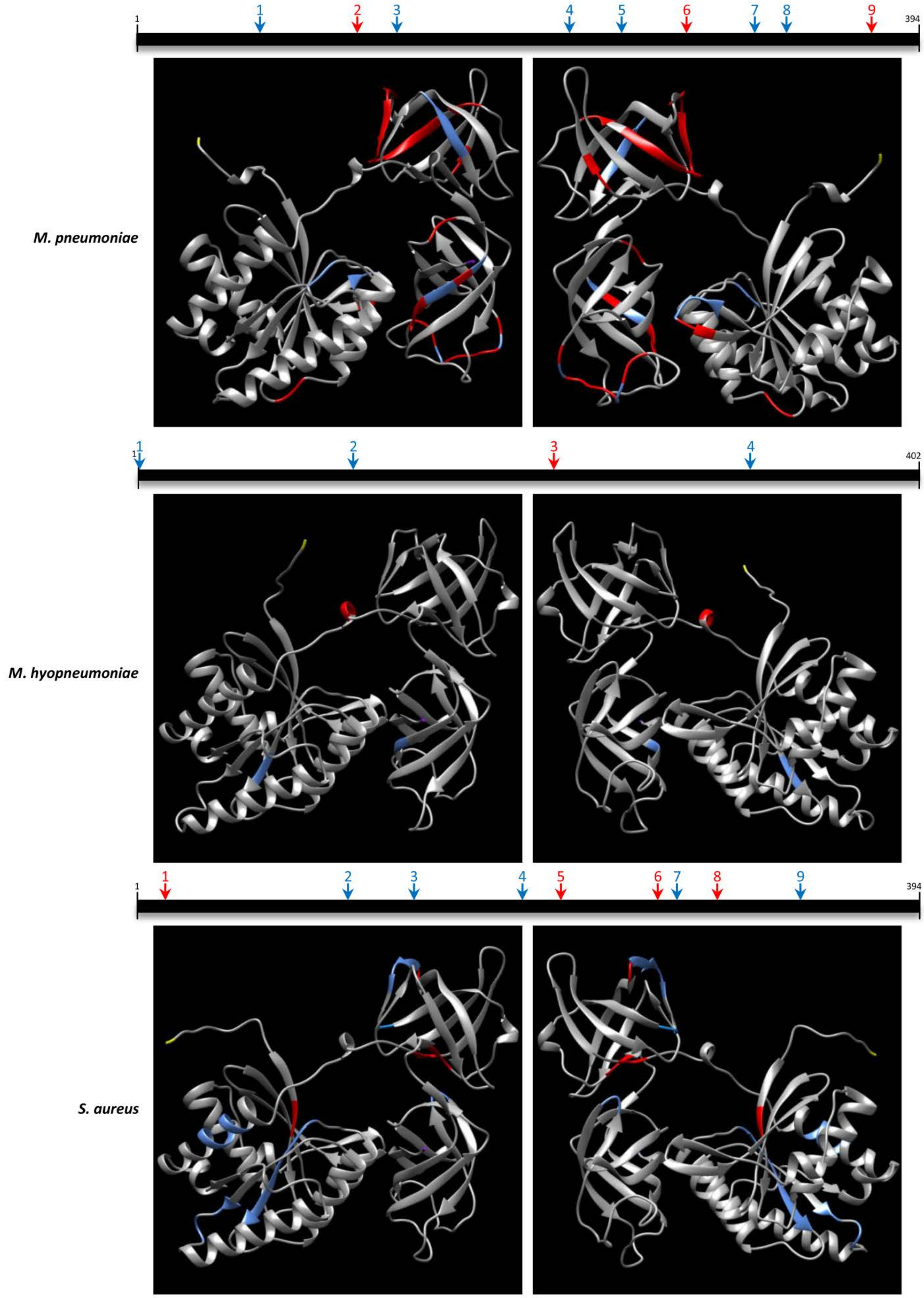

1
2
3
4
5
6
7
8
9
394
M. pneumoniae
1
2
3
4
402
M. hyopneumoniae
1
2
3
4
5
6
7
8
9
394
S. aureus

Figure S2. Predicted 3D ribbon structures of Mpn$_{\text{Ef-Tu}}$, Mhp$_{\text{Ef-Tu}}$ and Sa$_{\text{Ef-Tu}}$. Ef-Tu molecules are represented as black bars with identified cleavage sites displayed as arrows (blue, dimethyl labelling and red, semi-tryptic) with numbers to indicate each distinct cleavage site. Cleavage sites can also be seen in the ribbon structures as blue and red sections for dimethyl labelling and semi-tryptic sites, respectively. Images are reversed sides of each structure. Structures were predicted by MODELLER [97] and were based on *E. coli* Ef-Tu homologues: PDB: 4G5G_A (*M. pneumoniae*), PDB: 1DG1_H (*M. hyopneumoniae*) and PDB: 1DG1_H (*S. aureus*). For context, the N-terminus of the protein is shown in yellow, and the C-terminus in purple.

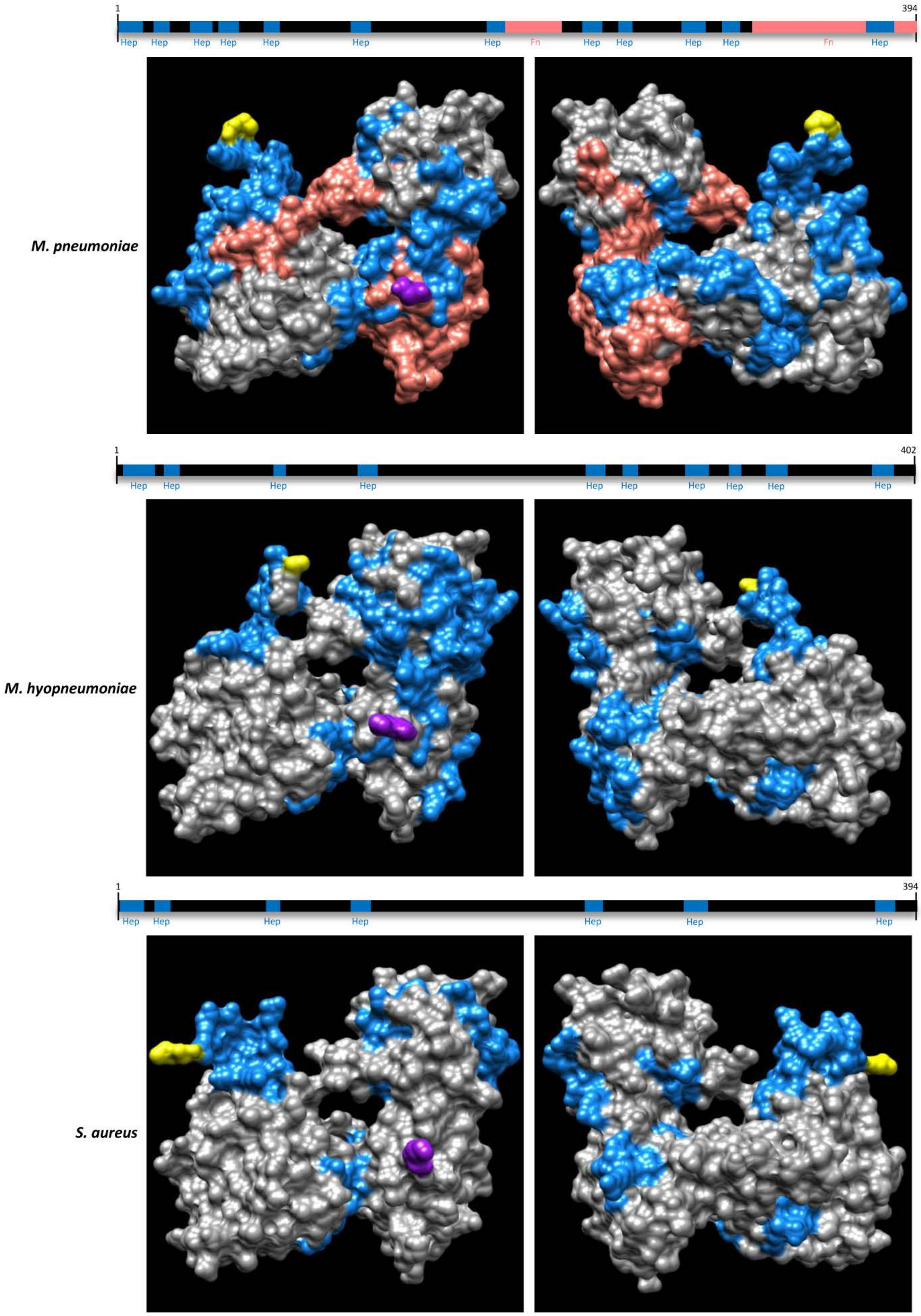
1
394
Hep Hep Hep Hep Hep Hep Hep Fn Hep Hep Hep Hep Fn Hep
M. pneumoniae
1
402
Hep Hep Hep Hep Hep Hep Hep Hep Hep Hep
M. hyopneumoniae
1
394
Hep Hep Hep Hep Hep Hep Hep
S. aureus

Figure S3. Predicted 3D space-filling structures for all three pathogens. Full length Ef-Tu are represented as black bars with predicted heparin-binding sites for each pathogen shown in blue boxes and regions in the structures. The two published fibronectin-binding domains for *M. pneumoniae* have also been mapped. Structures were predicted by MODELLER and were based on *E. coli* Ef-Tu homologues: PDB: 4G5G_A (*M. pneumoniae*), PDB: 1DG1_H (*M. hyopneumoniae*) and PDB: 1DG1_H (*S. aureus*). The N-terminus of the protein is shown in yellow, and the C-terminus in purple.

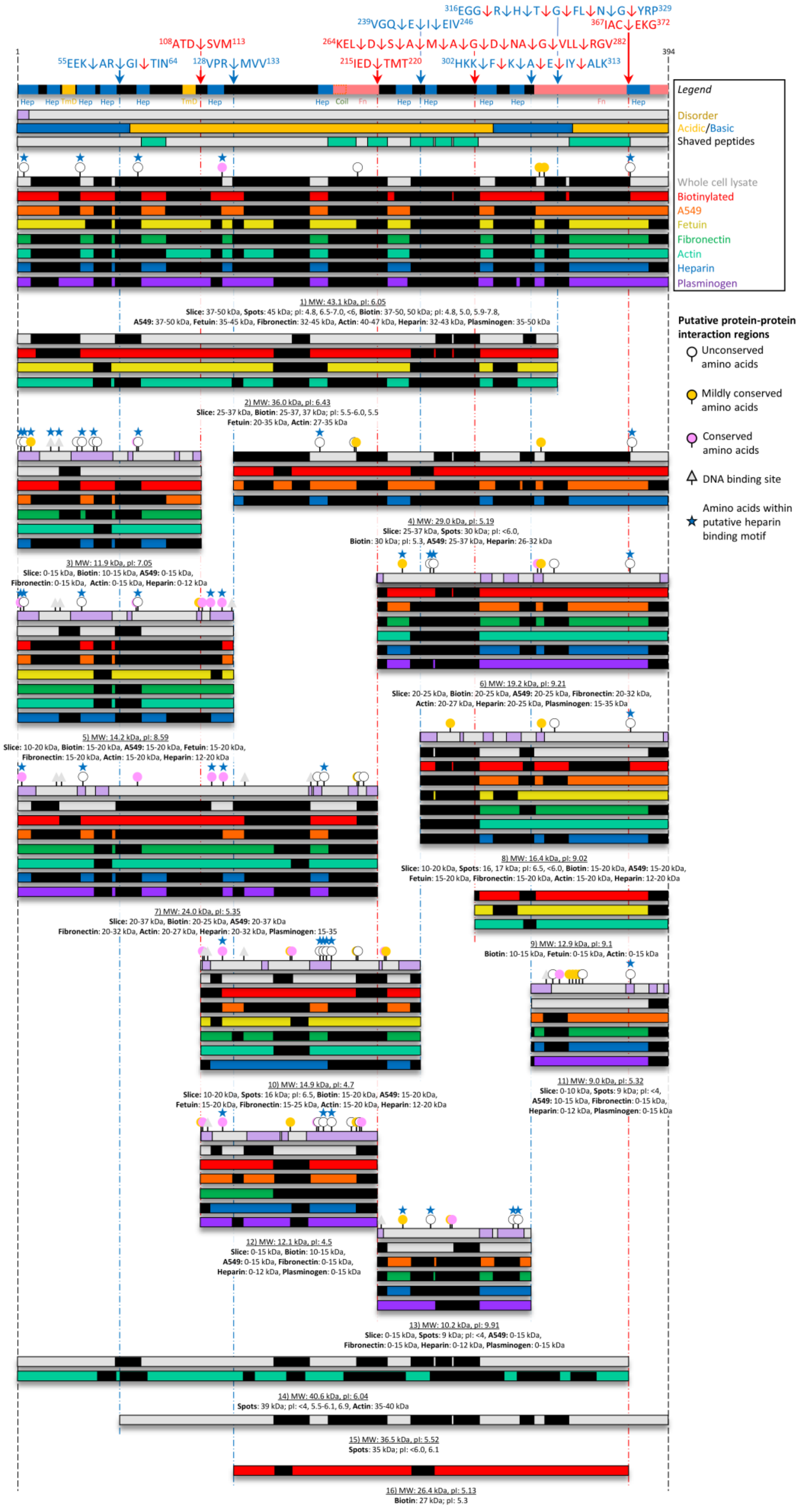

[108]ATD↓SVM[113]
[316]EGG↓R↓H↓T↓G↓FL↓N↓G↓YRP[329]
[239]VGQ↓E↓I↓EIV[246]
[367]IAC↓EKG[372]
[264]KEL↓D↓S↓A↓M↓A↓G↓D↓NA↓G↓VLL↓RGV[282]
1
394
[55]EEK↓AR↓GI↓TIN[64]
[128]VPR↓MVV[133]
[215]IED↓TMT[220]
[302]HKK↓F↓K↓A↓E↓IY↓ALK[313]
Hep
TmD
Coil
Fn
Legend
Disorder
Acidic/Basic
Shaved peptides
Whole cell lysate
Biotinylated
A549
Fetuin
Fibronectin
Actin
Heparin
Plasminogen
1) MW: 43.1 kDa, pI: 6.05
Slice: 37-50 kDa, Spots: 45 kDa; pI: 4.8, 6.5-7.0, <6, Biotin: 37-50, 50 kDa; pI: 4.8, 5.0, 5.9-7.8,
A549: 37-50 kDa, Fetuin: 35-45 kDa, Fibronectin: 32-45 kDa, Actin: 40-47 kDa, Heparin: 32-43 kDa, Plasminogen: 35-50 kDa
Putative protein-protein interaction regions
Unconserved amino acids
Mildly conserved amino acids
Conserved amino acids
DNA binding site
Amino acids within putative heparin binding motif
2) MW: 36.0 kDa, pI: 6.43
Slice: 25-37 kDa, Biotin: 25-37, 37 kDa; pI: 5.5-6.0, 5.5
Fetuin: 20-35 kDa, Actin: 27-35 kDa
4) MW: 29.0 kDa, pI: 5.19
Slice: 25-37 kDa, Spots: 30 kDa; pI: <6.0,
Biotin: 30 kDa; pI: 5.3, A549: 25-37 kDa, Heparin: 26-32 kDa
3) MW: 11.9 kDa, pI: 7.05
Slice: 0-15 kDa, Biotin: 10-15 kDa, A549: 0-15 kDa,
Fibronectin: 0-15 kDa, Actin: 0-15 kDa, Heparin: 0-12 kDa
6) MW: 19.2 kDa, pI: 9.21
Slice: 20-25 kDa, Biotin: 20-25 kDa, A549: 20-25 kDa, Fibronectin: 20-32 kDa,
Actin: 20-27 kDa, Heparin: 20-25 kDa, Plasminogen: 15-35 kDa
5) MW: 14.2 kDa, pI: 8.59
Slice: 10-20 kDa, Biotin: 15-20 kDa, A549: 15-20 kDa, Fetuin: 15-20 kDa,
Fibronectin: 15-20 kDa, Actin: 15-20 kDa, Heparin: 12-20 kDa
8) MW: 16.4 kDa, pI: 9.02
Slice: 10-20 kDa, Spots: 16, 17 kDa; pI: 6.5, <6.0, Biotin: 15-20 kDa, A549: 15-20 kDa,
Fetuin: 15-20 kDa, Fibronectin: 15-20 kDa, Actin: 15-20 kDa, Heparin: 12-20 kDa
7) MW: 24.0 kDa, pI: 5.35
Slice: 20-37 kDa, Biotin: 20-25 kDa, A549: 20-37 kDa
Fibronectin: 20-32 kDa, Actin: 20-27 kDa, Heparin: 20-32 kDa, Plasminogen: 15-35
9) MW: 12.9 kDa, pI: 9.1
Biotin: 10-15 kDa, Fetuin: 0-15 kDa, Actin: 0-15 kDa
10) MW: 14.9 kDa, pI: 4.7
Slice: 10-20 kDa, Spots: 16 kDa; pI: 6.5, Biotin: 15-20 kDa, A549: 15-20 kDa,
Fetuin: 15-20 kDa, Fibronectin: 15-25 kDa, Actin: 15-20 kDa, Heparin: 12-20 kDa
11) MW: 9.0 kDa, pI: 5.32
Slice: 0-10 kDa, Spots: 9 kDa; pI: <4,
A549: 10-15 kDa, Fibronectin: 0-15 kDa,
Heparin: 0-12 kDa, Plasminogen: 0-15 kDa
12) MW: 12.1 kDa, pI: 4.5
Slice: 0-15 kDa, Biotin: 10-15 kDa,
A549: 0-15 kDa, Fibronectin: 0-15 kDa,
Heparin: 0-12 kDa, Plasminogen: 0-15 kDa
13) MW: 10.2 kDa, pI: 9.91
Slice: 0-15 kDa, Spots: 9 kDa; pI: <4, A549: 0-15 kDa,
Fibronectin: 0-15 kDa, Heparin: 0-12 kDa, Plasminogen: 0-15 kDa
14) MW: 40.6 kDa, pI: 6.04
Spots: 39 kDa; pI: <4, 5.5-6.1, 6.9, Actin: 35-40 kDa
15) MW: 36.5 kDa, pI: 5.52
Spots: 35 kDa; pI: <6.0, 6.1
16) MW: 26.4 kDa, pI: 5.13
Biotin: 27 kDa; pI: 5.3

Figure S4. Cleavage map of Mpn$_{Ef-Tu}$. Peptides (black boxes in coloured bars) identified by mass spectrometry of affinity chromatography of A549 surface proteins (orange bars), fetuin (yellow bars), fibronectin (green bars), actin (teal bars) and plasminogen (purple bars). Circles and triangles fragments indicate amino acid binding sites with either proteins or DNA, respectively. Purple boxes within grey bars are disordered regions within fragments. Red bars indicate peptides identified from surface biotinylation and peptides released from trypsin surface shaving can be seen as the green boxes in the grey bar above the full length protein.

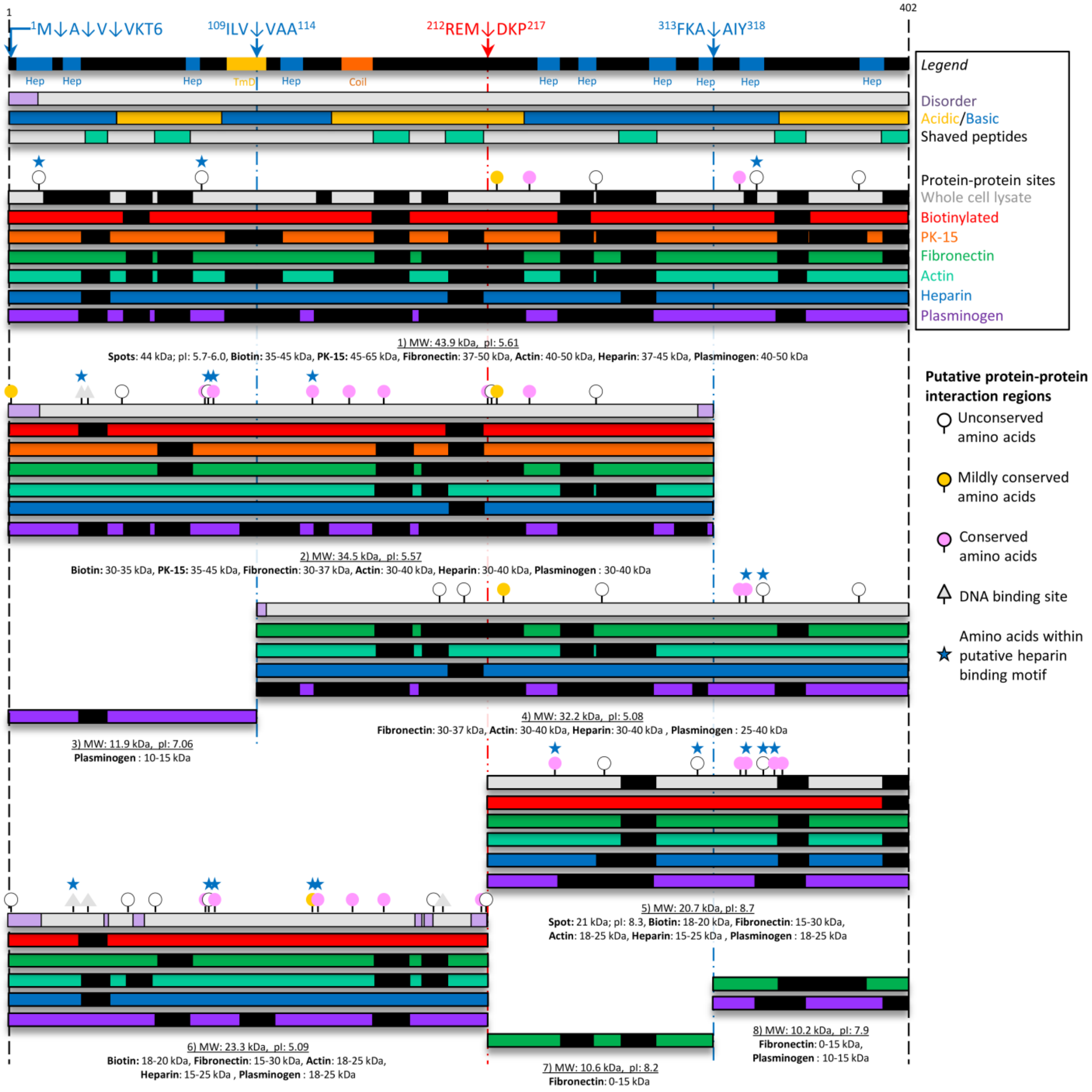

Figure S5. Cleavage map of Mhp$_{\text{Ef-Tu}}$. Peptides (black boxes in coloured bars) identified by mass spectrometry of affinity chromatography of PK-15 surface proteins (orange bars), fibronectin (green bars), actin (teal bars) and plasminogen (purple bars). Circles and triangles fragments indicate amino acid binding sites with either proteins or DNA, respectively. Purple boxes within grey bars are disordered regions within fragments. Red bars indicate peptides

identified from surface biotinylation and peptides released from trypsin surface shaving can be seen as the green boxes in the grey bar above the full length protein.

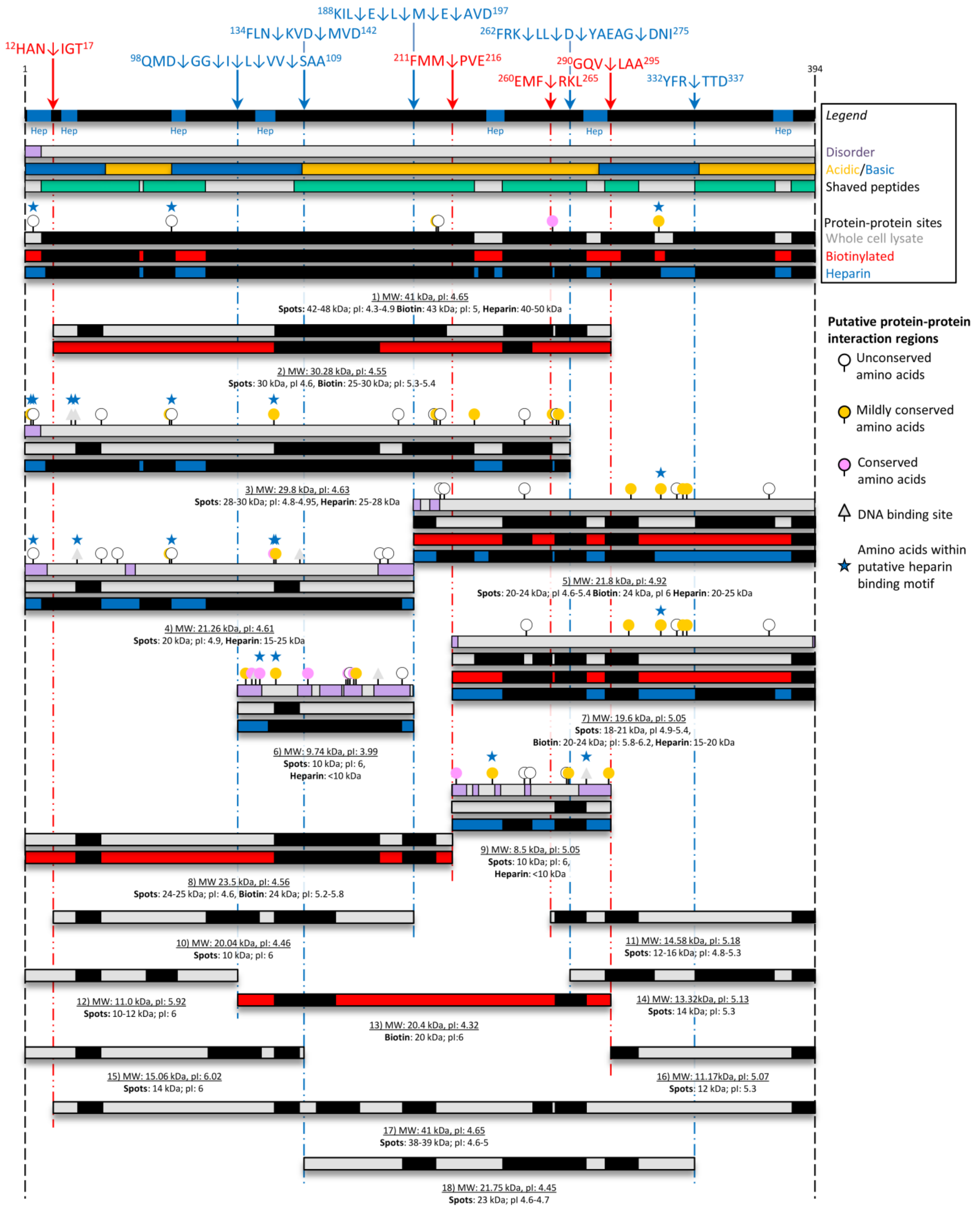

Figure S6. Cleavage map of Ef-Tu$_{Sa}$. An expansion of the cleavage maps in Figure 2. Includes peptides (black boxes in coloured bars) identified by mass spectrometry of Ef-Tu fragments that

don’t bind heparin or not on the surface (Fragments 10 – 18). Circles and triangles fragments indicate amino acid binding sites with either proteins or DNA, respectively. Purple boxes within grey bars are disordered regions within fragments. Red bars indicate peptides identified from surface biotinylation and peptides released from trypsin surface shaving can be seen as the green boxes in the grey bar above the full length protein.

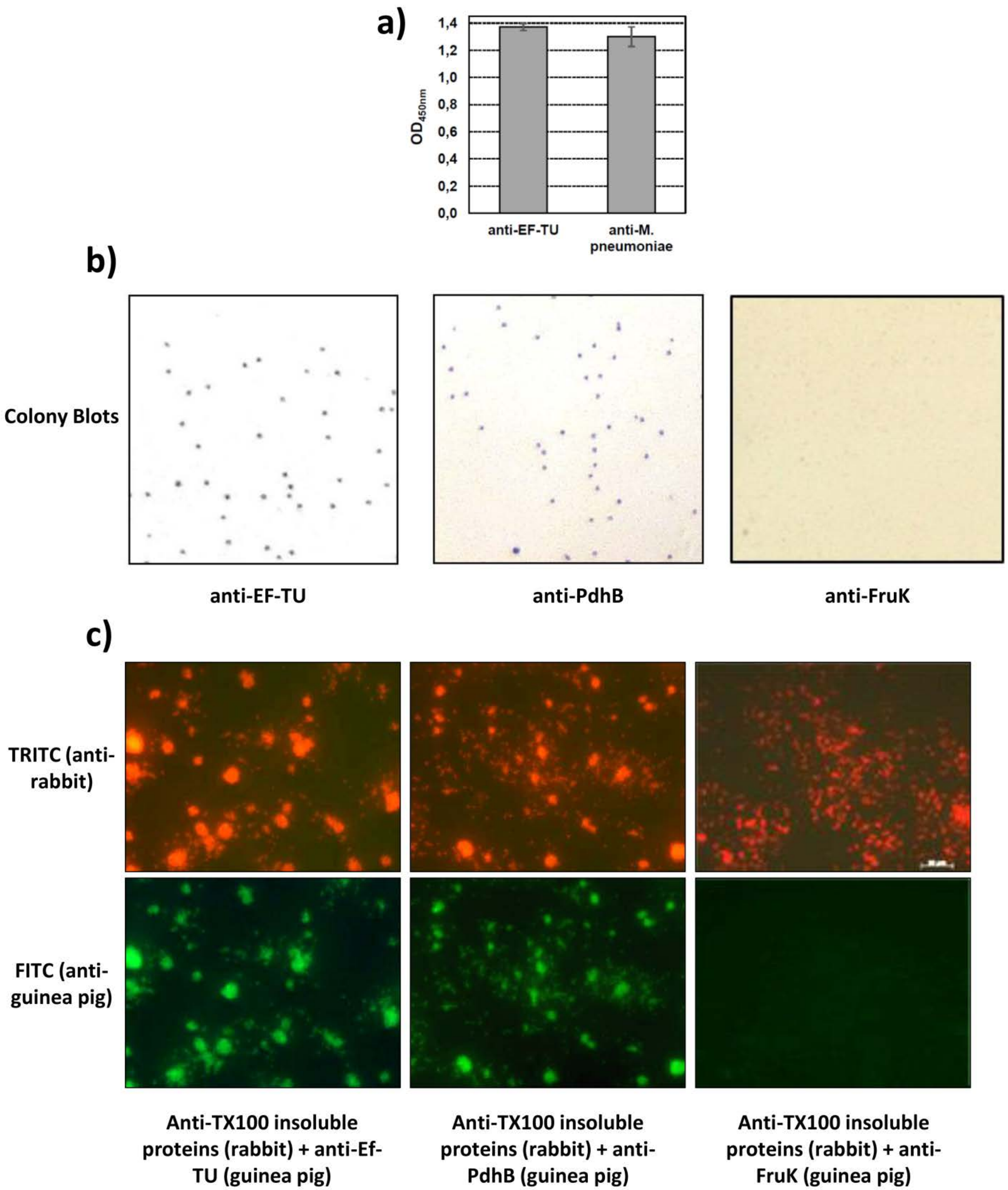

Figure S7: Mpn$_{Ef\text{-}Tu}$ resides on the surface of *M. pneumoniae*. A) Anti - rMpn$_{Ef\text{-}Tu}$ antibodies recognise *M. pneumoniae* cells in a whole cell ELISA assay; eight replicates. B) Colony blots of *M. pneumoniae* probed with anti - rMpn$_{Ef\text{-}Tu}$ antibodies, PdhB (positive control) and 1-

phosphofructokinase (negative control). C) Immunofluorescence microscopy of *M. pneumoniae* cells probed with antibodies against TX100 insoluble proteins (TRITC; cell control), Ef-Tu, PdhB (positive control) and 1-phosphofructokinase as the negative control (FITC).

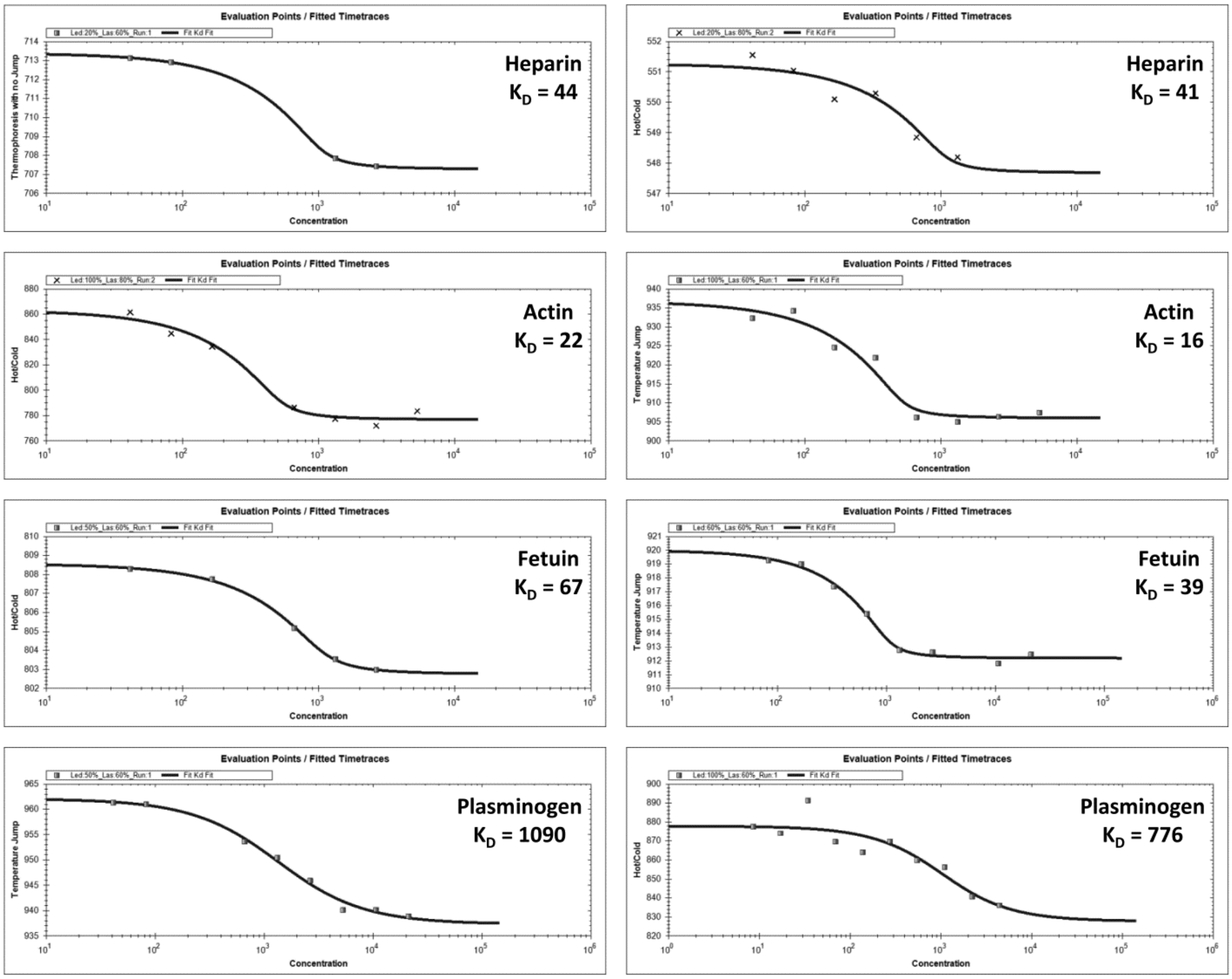

Figure S8: Microscale thermophoresis output depicting the interaction of rMpn$_{\text{Ef-Tu}}$ with human molecules. Concentration of rMpn$_{\text{Ef-Tu}}$ is plotted against thermophoretic movement of fluorescent human molecules. Experiments performed in duplicate, each panel representing one replicate.

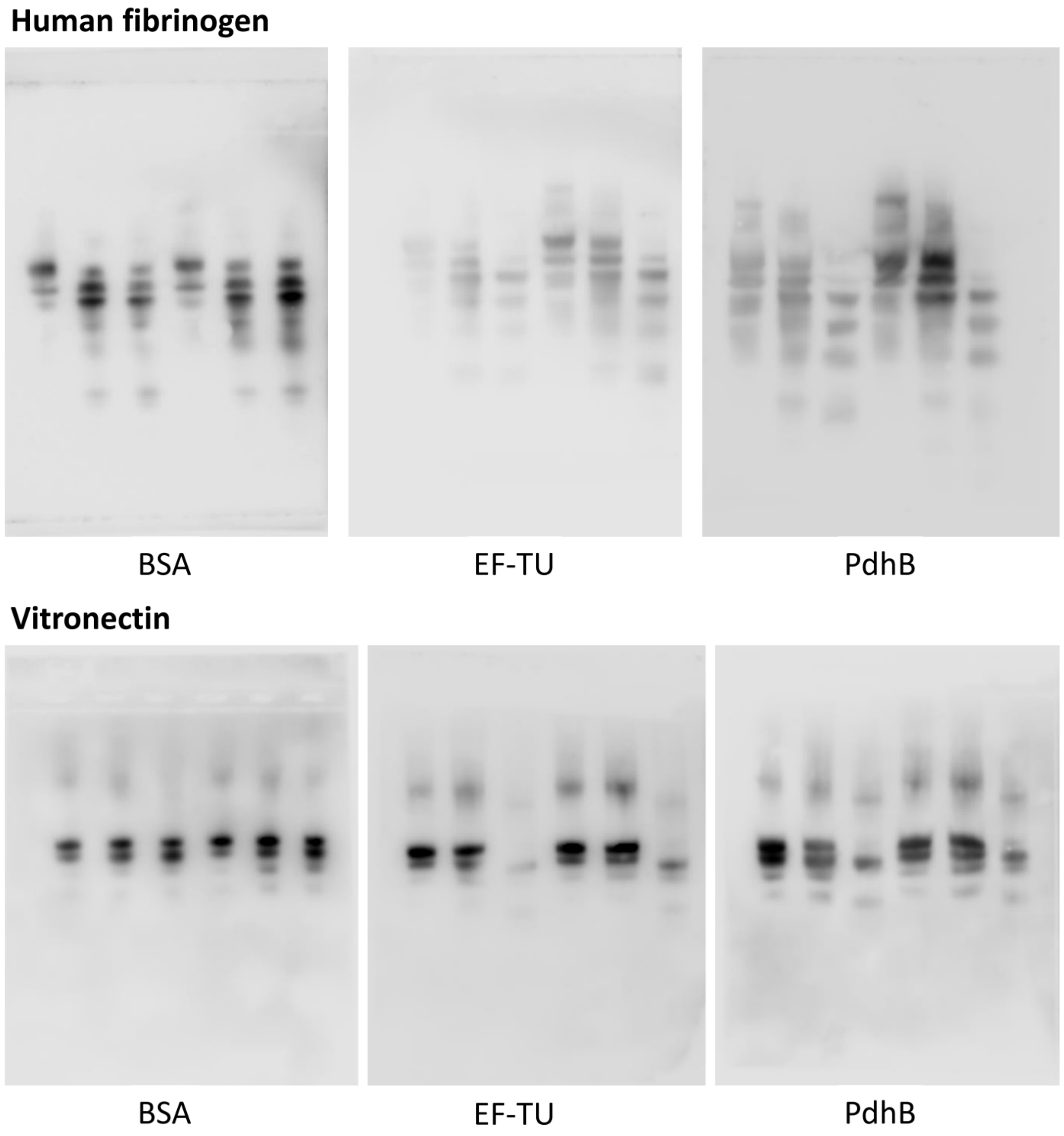

Figure S9: Full length blots showing degradation of human fibrinogen and vitronectin. Contrast adjusted (increased) to demonstrate no additional bands in full blots. Corresponding to Figure 5B, degradation with plasminogen activated by either urinary plasminogen activator or tissue plasminogen activator. Contrast and brightness editing are equal across the blots.

Will be presented as “Supplementary file S8- Tables”

Table S1. Putative heparin-binding motifs identified in $Mpn_{Ef\text{-}Tu}$, $Mhp_{Ef\text{-}Tu}$, and $Sa_{Ef\text{-}Tu}$.

**Amino acid range**	**Sequence**
	**$Mpn_{Ef\text{-}Tu}$ (Uniprot #: P23568)**
2 – 13:	aReKfdRsKpHv
19 – 26:	gHidHgKt=
37 – 47:	aKegKsaatRy
51 – 60:	dKapeeKaRg
73 – 80:	dKRHyaHv*
116 – 125:	tReHillaRq=
183 – 191:	pKweaKiHd
230 – 239:	gRveRgelKv=
248 – 254:	lRpiRKa
279 – 290:	lRgvdRKeveRg
299 – 307:	iKpH*KKfK*a
370 – 383:	eKgsKfsiReggRt=
	**$Mhp_{Ef\text{-}Tu}$ (Uniprot #: Q4A9G1)**
4 – 19:	vKttgKKdfdRsKeHi
25 – 32:	gHvdHgKt=
79 – 86:	dKRHyaHv*
122 – 131:	tReHillsKq=
237 – 246:	gKveRgqvKl=
255 – 262:	yReepKKt
287 – 298:	lRgvdrKdieRg
302 – 315:	aKpKtiipHtKfKa
327 – 337:	gRHtpffKnyKp
381 – 391:	tKfsiReggRt=
	**$Sa_{Ef\text{-}Tu}$ (Uniprot #: Q2G0N0)**
2 – 13:	aKeKfdRsKeHa
19 – 26:	gHvdHgKt=
73 – 80:	dKRHyaHv*
116 – 125:	tReHillsRn=
230 – 239:	gRveRgqiKv=
279 – 290:	lRgvaRedvqRg
373 – 383:	tRfsiReggRt=

$Mpn_{Ef\text{-}Tu}$, $Mhp_{Ef\text{-}Tu}$, and $Sa_{Ef\text{-}Tu}$ were searched for patters x-[HKR]-x(0,2)-[HKR]-x(0,2)-[HKR]-x and x-[HKR]-x(1,4)-[HKR]-x(1,4)-[HKR]-x using ScanProsite [144]. * indicates the motif (dKRHyaH) which is found in all three pathogens. = indicates the motifs that are highly homologous (up to three non-basic residues different) in all three pathogens.

Table S2A. Number of binding sites in full length and fragments of $\mathrm{Mpn}_{\mathrm{Ef\text{-}Tu}}$.

Analysis of the $\mathrm{Mpn}_{\mathrm{Ef\text{-}Tu}}$ for the putative protein-protein (P:P) and protein-nucleic acid interaction sites using ISIS [95].

$\mathrm{Mpn}_{\mathrm{Ef\text{-}Tu}}$ (Uniprot #: P23568)

**Fragment number**	**Full length**	**3**	**4**	**5**	**6**	**7**	**8**	**10**	**11**	**12**	**13**
**Range (amino acid)**	1-394	1-110	131-394	1-130	218-394	1-217	244-394	111-242	308-394	111-217	218-307
**Exposed P:P**	8	15	5	13	10	17	6	12	9	19	11
**Buried P:P**	6	5	2	7	5	2	5	3	6	2	0
**DNA-binding**	0	8	0	5	0	6	0	3	2	1	1

Table S2B. Number of binding sites in full length and fragments of $\mathrm{Mhp}_{\mathrm{Ef\text{-}Tu}}$.

Analysis of the $\mathrm{Mhp}_{\mathrm{Ef\text{-}Tu}}$ for the putative protein-protein (P:P) and protein-nucleic acid interaction sites using ISIS [95].

$\mathrm{Mhp}_{\mathrm{Ef\text{-}Tu}}$ (Uniprot #: Q4A9G1)

**Fragment number**	**Full length**	**1**	**4**	**5**	**6**
**Range (amino acid)**	Full	1-315	112-402	215-402	1-215
**Exposed P:P**	10	13	9	10	18
**Buried P:P**	2	3	1	3	4
**DNA-binding**	0	3	0	0	4

Table S2C. Number of binding sites in full length and fragments of $\mathrm{Sa}_{\mathrm{Ef\text{-}Tu}}$.

Analysis of the $Sa_{Ef\text{-}Tu}$ for the putative protein-protein (P:P) and protein-nucleic acid interaction sites using ISIS [95].

$Sa_{Ef\text{-}Tu}$ (Uniprot #: Q2G0N0)

**Fragment number**	**Full length**	**3**	**4**	**5**	**6**	**7**	**9**
**Range (amino acid)**	**Full**	**1-266**	**1-192**	**193-394**	**104-192**	**214-394**	**214-292**
**Exposed P:P**	10	13	10	7	12	5	10
**Buried P:P**	6	12	6	5	2	5	1
**DNA-binding**	0	3	4	0	2	0	1

Will be presented as “Supplementary file S9- Bioinformatics”

Supplementary Materials - Bioinformatics

Conserved/Non-conserved regions:

Analysis of conservation of amino acids in $Mpn_{Ef\text{-}Tu}$, $Mhp_{Ef\text{-}Tu}$, and $Sa_{Ef\text{-}Tu}$.

Degree of conservation in $Mpn_{Ef\text{-}Tu}$ was calculated by The ConSurf server [96]. Colours indicate degree of conservation of the amino acid across species. Red diamond sticks indicate predicted protein binding sites by ISIS [95,149]. Grey, yellow and purple circle sticks indicate predicted nucleotide, DNA and RNA binding regions, respectively by SomeNA [149,150]. Below the sticks are three rows: i) Blue and red bars indicate predicted beta-strand and helix secondary structures, respectively by REPROFSec [147,149], ii) Blue and yellow bars indicate predicted regions that are exposed and buried to solvent accessibilities, respectively by PROFAcc [147,149]. iii) Green bars indicate predicted disordered regions by Meta-Disorder (MD) [146,149]. Circled amino acids represent predicted binding sites. Amino acids indicated by a triangle indicate predicted nucleotide binding sites.

<u>Legend:</u>

The conservation scale:

1	2	3	4	5	6	7	8	9

Variable Average Conserved

e - An exposed residue according to the neural-network algorithm.
b - A buried residue according to the neural-network algorithm.
f - A predicted functional residue (highly conserved and exposed).
s - A predicted structural residue (highly conserved and buried).
X - Insufficient data - the calculation for this site was performed on less than 10% of the sequences.

Mpn$_{\text{Ef-Tu}}$ (Uniprot #: P23568)

Full length protein: 1-394

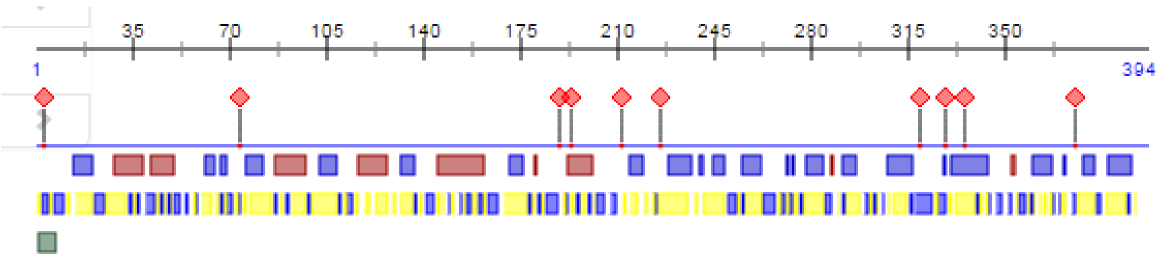

1	11	21	31	41
MAREKFDRSK	PHVNVGTIGH	IDHGKTTLTA	AICTVLAKEG	KSAATRYDQI
51	61	71	81	91
DKAPEEKARG	ITINSAHVEY	SSDKRHYAHV	DCPGHADYIK	NMITGAAQMD
101	111	121	131	141
GAILVVSATD	SVMPQTREHI	LLARQVGVPR	MVVFLNKCDI	ATDEEVQELV
151	161	171	181	191
AEEVRDLLTS	YGFDGKNTPI	IYGSALKALE	GDPKWEAKIH	DLMNAVDEWI
201	211	221	231	241
PTPEREVDKP	FLLAIEDTMT	ITGRGTVVTG	RVERGELKVG	QEIEIVGLRP
251	261	271	281	291
IRKAVVTGIE	MFKKELDSAM	AGDNAGVLLR	GVDRKEVERG	QVLAKPGSIK
301	311	321	331	341
PHKKFKAEIY	ALKKEEGGRH	TGFLNGYRPQ	FYFRTTDVTG	SISLPENTEM
351	361	371	381	391
VLPGDNTSIT	VELIAPIACE	KGSKFSIREG	GRTVGAGSVT	EVLE

Fragment 3: 1-110

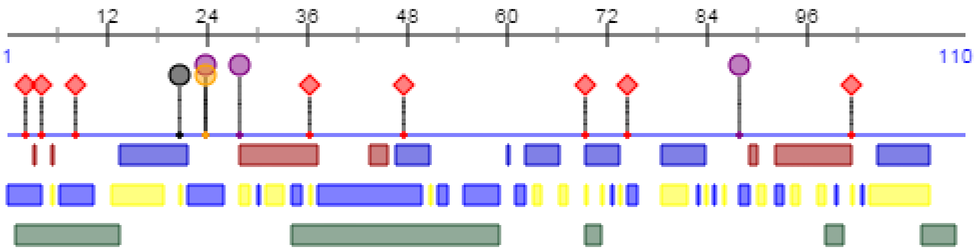

1 MAREKFDRSK	11 PHVNVGTIGH	21 IDHGKTTLTA	31 AICTVLAKEG	41 KSAATRYDQI
51 DKAPEEKARG	61 ITINSAHVEY	71 SSDKRHYAHV	81 DCPGHADYIK	91 NMITGAAQMD
101 GAILVVSATD				

Fragment 4: 131-394

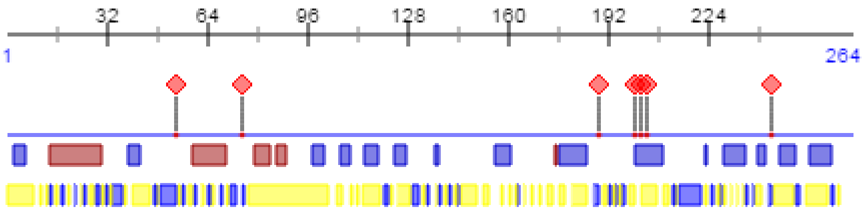

131 MVVFLNKCDI	141 ATDEEVQELV	151 AEEVRDLLTS	161 YGFDGKNTPI	171 IYGSALKALE
181 GDPKWEAKIH	191 DLMNAVDEWI	201 PTPEREVDKP	211 FLLAIEDTMT	221 ITGRGTVVTG
231 RVERGELKVG	241 QEIEIVGLRP	251 IRKAVVTGIE	261 MFKKELDSAM	271 AGDNAGVLLR
281 GVDRKEVERG	291 QVLAKPGSIK	301 PHKKFKAEIY	311 ALKKEEGGRH	321 TGFLNGYRPQ
331 FYFRTTDVTG	341 SISLPENTEM	351 VLPGDNTSIT	361 VELIAPIACE	371 KGSKFSIREG
381 GRTVGAGSVT	391 EVLE			

Fragment 5: 1-130

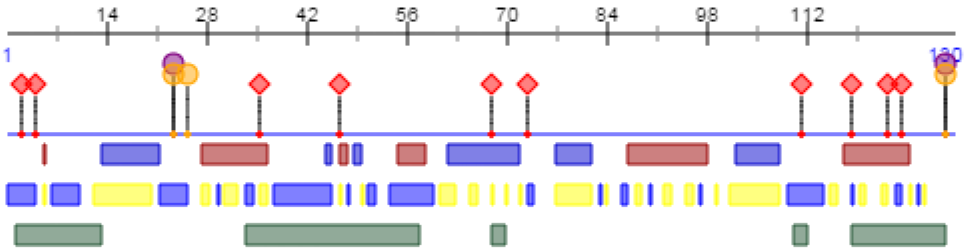

1 MAREKFDRSK	11 PHVNVGTIGH	21 IDHGKTTLTA	31 AICTVLAKEG	41 KSAATRYDQI
51 DKAPEEKARG	61 ITINSAHVEY	71 SSDKRHYAHV	81 DCPGHADYIK	91 NMITGAAQMD
101 GAILVVSATD	111 SVMPQTREHI	121 LLARQVGVPR		

Fragment 6: 218-394

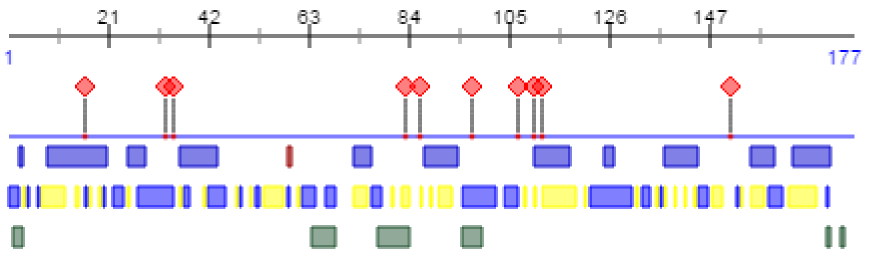

218 TMTITGRGTV	228 VTGRVERGEL	238 KVGQEIEIVG	248 LRPIRKAVVT	258 GIEMFKKELD
268 SAMAGDNAGV	278 LLRGVDRKEV	288 ERGQVLAKPG	298 SIKHKKFKA	308 EIYALKKEEG
318 GRHTGFLNGY	328 RPQFYFRTTD	338 VTGSISLPEN	348 TEMVLPGDNT	358 SITVELIAPI
368 ACEKGSKFSI	378 REGGRTVGAG	388 SVTEVLPE		

Fragment 7: 1-217

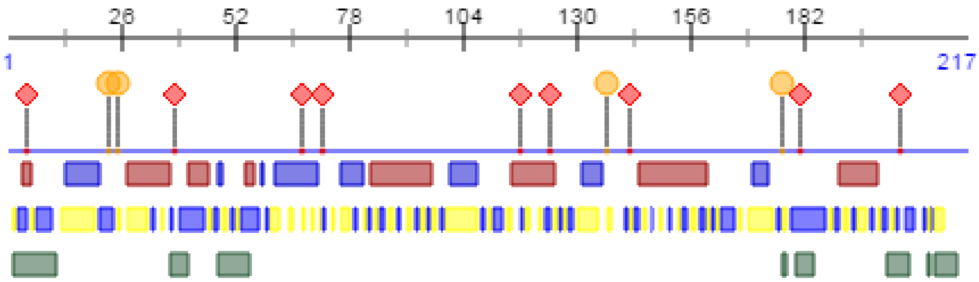

1 MAREKFDRSK	11 PHVNVGTIGH	21 IDHGKTTLTA	31 AICTVLAKEG	41 KSAATRYDQI

51	61	71	81	91
DKAPEEKARG	ITINSAHVEY	SSDKRHYAHV	DCPGHADYIK	NMITGAAQMD
101	111	121	131	141
GAILVVSATD	SVMPQTREHI	LLARQVGVPR	MVVFLNKCDI	ATDEEVQELV
151	161	171	181	191
AEEVRDLLTS	YGFDGKNTPI	IYGSALKALE	GDPKWEAKIH	DLMNAVDEWI
201	211			
PTPEREVDKP	FLLAIED			

Fragment 8: 244-394

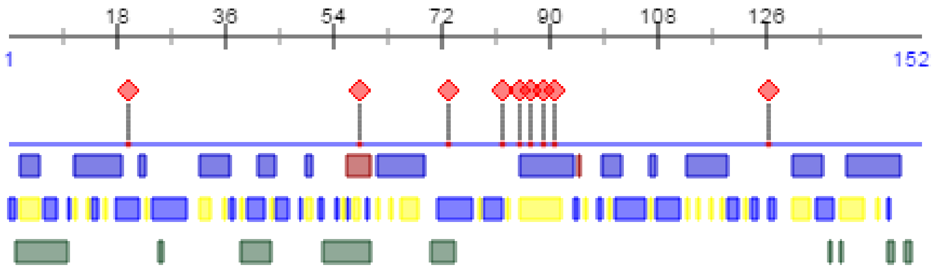

243	253	263	273	283
IEIVGLRPIR	KAVVTGIEMF	KKELDSAMAG	DNAGVLLRGV	DRKEVERGQV
293	303	313	323	333
LAKPGSIKPH	KKFKAEIYAL	KKEEGGRHTG	FLNGYRPQFY	FRTTDVTGSI
343	353	363	373	383
SLPENTEMVL	PGDNTSITVE	LIAPIACEKG	SKFSIREGGR	TVGAGSVTEV
393				
LE				

Fragment 10: 111-242

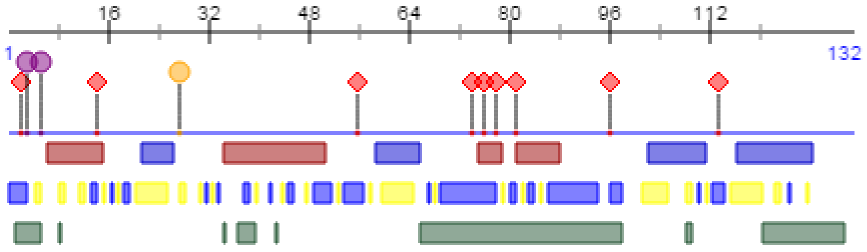

111 SVMPQTREHI	121 LLARQVGVPR	131 MVVFLNKCDI	141 ATDEEVQELV	151 AEEVRDLLTS
161 YGFDGKNTPI	171 IYGSALKALE	181 GDPKWEAKIH	191 DLMNAVDEWI	201 PTPEREVDKP
211 FLLAIEDTMT	221 ITGRGTVVTG	231 RVERGELKVG	QE	

Fragment 11: 308-391

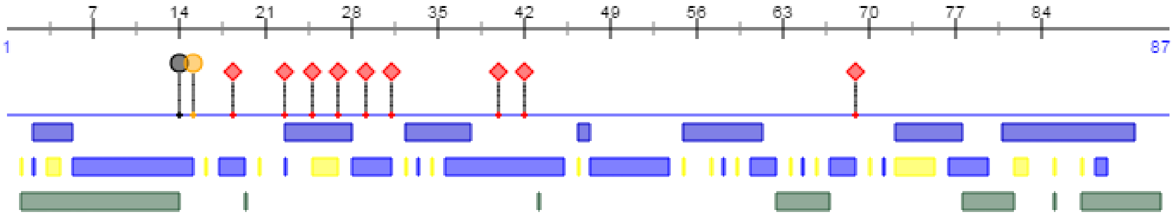

308 EIYALKKEEG	318 GRHTGFLNGY	328 RPQFYFRTTD	338 VTGSISLPEN	348 TEMVLPGDNT
358 SITVELIAPI	368 ACEKGSKFSI	378 REGGRTVGAG	388 SVTEVLE	

Fragment 12: 111-217

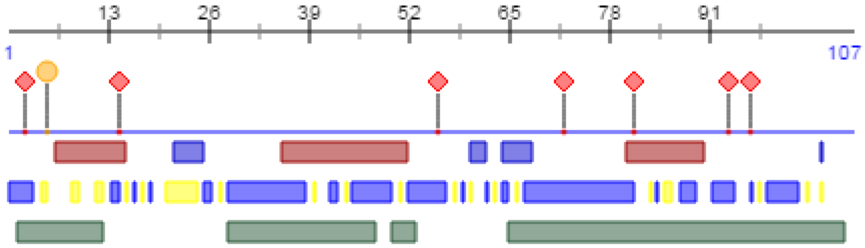

111 SVMPQTREHI	121 LLARQVGVPR	131 MVVFLNKCDI	141 ATDEEVQELV	151 AEEVRDLLTS
161 YGFDGKNTPI	171 IYGSALKALE	181 GDPKWEAKIH	191 DLMNAVDEWI	201 PTPEREVDKP
211 FLLAIED				

Fragment 13: 218-307

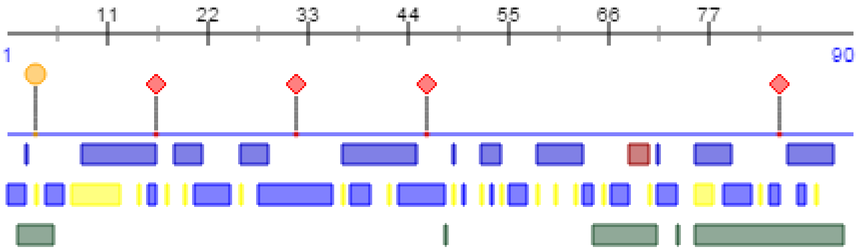

218	228	238	248	258
TMTITGRGTV	VTGRVERGEL	KVGQEIEIVG	LRPIRKAVVT	GIEMFKKELD
268	278	288	298	
SAMAGDNAGV	LLRGVDRKEV	ERGQVLAKPG	SIKPHKKFKA	

Mhp$_{Ef\text{-}Tu}$ (Uniprot #: Q4A9G1)

Full length protein: 1-402

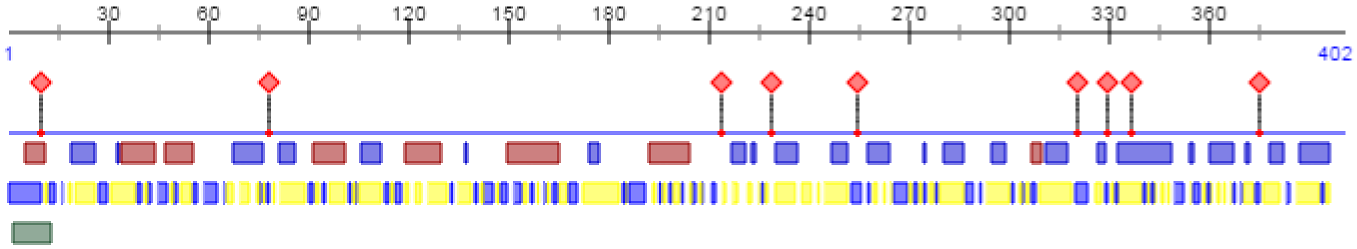


```
1           11          21          31          41
MAVVKTTGKK  DFDRSKEHIN  IGTIGHVDHG  KTTLTAAIST  VLAKRGLAEA
eebeeebbee  ebeeeeeebb  bbbbbbbeeb  ebebebbbbe  bbbeeeeeeb
      s f      f f f s   sssss ffs  fsfsf ss

51          61          71          81          91
KDYASIDAAP  EEKARGITIN  TAHIEYSTDK  RHYAHVDCPG  HADYIKNMIT
eebeebeebe  eeeeebbbbb  bbebebeeee  ebbbbbbbee  ebebbeebbb
     sf sf  ff  fssss     f fs  f   fssss s ff  fsfs ffsss

101         111         121         131         141
GAAQMDGAIL  VVAATDGPMP  QTREHILLSK  QVGVPKMVVF  LNKIDLLEGE
bbbbbebbbb  bbbbeeeeee  ebeebbbbbe  ebebebbbbb  bbebeeeeee
sssssfsss   s  s fffff  fsffs ss    fs sf   s    sf f f  f

151         161         171         181         191
EEMVDLVEVE  IRELLSSYDF  DGDNTPIIRG  SARGALEGKP  EWEAKVLELM
eebbebbeee  beebbeeeeb  eeeebebbeb  bbbebbeeee  eeeeebeebb
 f   ssf f   f ss   f   fff  f   s  ss                  s

201         211         221         231         241
DAVDSYIDSP  VREMDKPFLM  AVEDVFTITG  RGTVATGKVE  RGQVKLNEEV
eebeeebeee  eeeeeeeebb  ebeebbbbbb  ebebbebebe  ebebebeeeb
  f   f f    f    fs s    ff   s s  fsfs fsf f  fs

251         261         271         281         291
EIVGYREEPK  KTVITGIEMF  NKNLQTAMAG  DNAGVLLRGV  DRKDIERGQV
ebbbbeeeee  eeebebbbeb  eebbeebeee  eebebbbebb  eeeebeebeb
f  s            f  fss   f sf   ff  ff f ssfs    f    fs s

301         311         321         331         341
IAKPKTIIPH  TKFKAAIYAL  KKEEGGRHTP  FFKNYKPQFY  FRTTDVTGGI
beeeeeeeee  eebebebbbb  eeeeeeeeee  ebeeeeeebb  bebeeeebeb
   f    ff         s s   f fffffff  f   ffffs    fsffffs
```

351 EFEPGREMVI ebeebbebbb s s s
361 PGDNVDLTVE eeeebebebe ffff
371 LIAPIAVEQG bbbbbbbeeb s s s
381 TKFSIREGGR bebbbeeeee sssffff
391 TVGAGTVTEI bbbbbebbeb sss s
401 IK ee

Fragment 1: 1-315

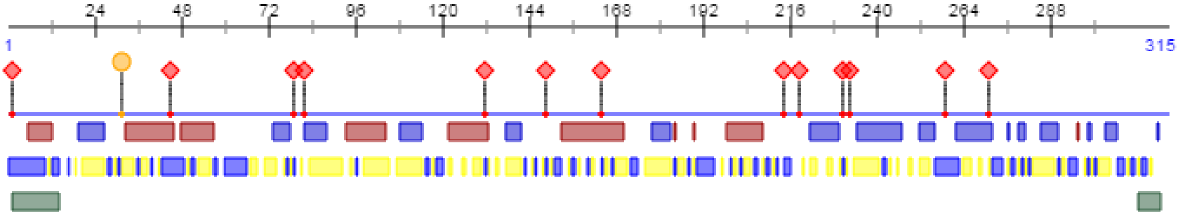

1 MAVVKTTGKK eebeeebbee ssf
11 DFDRSKEHIN ebeeeeeebb f f f s
21 IGTIGHVDHG bbbbbbbeeb sssss ffs
31 KTTLTAAIST ebebebbbbe fsfsfsss
41 VLAKRGLAEA bbbeeeeeeb

51 KDYASIDAAP eebeebeebe sf sf
61 EEKARGITIN eeeeebbbbb ff fssss
71 TAHIEYSTDK bbebebeeee f fs f
81 RHYAHVDCPG ebbbbbbbee fssss s ff
91 HADYIKNMIT ebebbeebbb fsfs ffsss

101 GAAQMDGAIL bbbbbebbbb ssssssfs s
111 VVAATDGPMP bbbbbeeeee s s ff ff
121 QTREHILLSK ebeebbbbbe fsffs ss f
131 QVGVPKMVVF ebebebbbbb fs sf s
141 LNKIDLLEGE bbebeeeeee sf f f f

151 EEMVDLVEVE eebbebbeee f ssfff
161 IRELLSSYDF beebbeeeeb f ss f
171 DGDNTPIIRG eeeebebbeb ff f
181 SARGALEGKP bbbebbeeee ss
191 EWEAKVLELM ebeeebeebb s

201 DAVDSYIDSP ebbeeebeee f f f
211 VREMDKPFLM eeeeeeebbb f fs s
221 AVEDVFTITG ebeebbbbbb ff s s
231 RGTVATGKVE ebebbebebe fsfs fsf f
241 RGQVKLNEEV ebebeeeeeb fs

251 EIVGYREEPK ebbbbeeeee
261 KTVITGIEMF eeebebbebb
271 NKNLQTAMAG eebbeebeee
281 DNAGVLLRGV eebebbbebb
291 DRKDIERGQV eeeebeebeb

```
  s           f  fss  f sf   ff ff f ssfs        fs s
301         311
IAKPKTIIPH  TKFKA
beeeeeeeee  eeeee
   f     f   f f
```

Fragment 4: 112-402

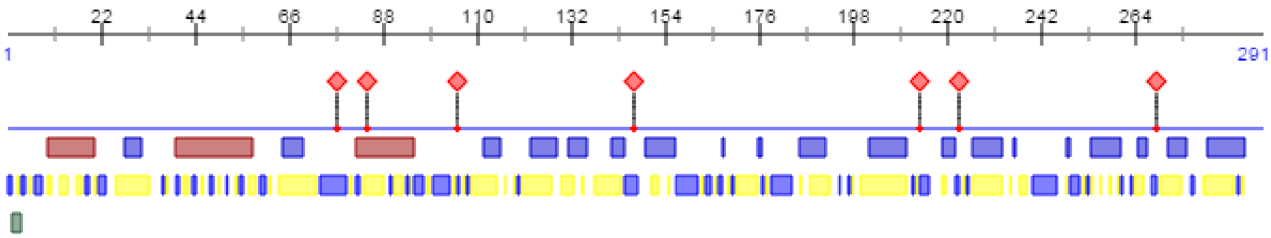


```
112         122         132         142         152
VAATDGPMPQ  TREHILLSKQ  VGVPKMVVFL  NKIDLLEGEE  EMVDLVEVEI
eeeeeeeeee  beebbbbbee  bebebbbbbb  bebeeeeeee  ebbebbeeeb
f f ffffff  s fs ss  f  s sf    s   sf f f  f   f   ssf f

162         172         182         192         202
RELLSSYDFD  GDNTPIIRGS  ARGALEGKPE  WEAKVLELMD  AVDSYIDSPV
eebbeeeebe  eeebebbebb  bbebbeeeee  beeebeebbe  bbeeebeeee
f ss        ff  f     s s  s               s      f   f f

212         222         232         242         252
REMDKPFLMA  VEDVFTITGR  GTVATGKVER  GQVKLNEEVE  IVGYREEPKK
eeeeeebbbe  beebbbbbbe  bebbebebee  bebebeeebe  bbbbeeeeee
f    fs s    ffs  s sf  sfs fsf ff  s        f    s

262         272         282         292         302
TVITGIEMFN  KNLQTAMAGD  NAGVLLRGVD  RKDIERGQVI  AKPKTIIPHT
eebebbebbe  ebbeebeeee  ebbbbbebbe  eeebeebebb  eeeeeeeeee
   f  fss   f sf    fff f s ssfs    f    fs s     f     ff

312         322         332         342         352
KFKAAIYALK  KEEGGRHTPF  FKNYKPQFYF  RTTDVTGGIE  FEPGREMVIP
ebebebbbbe  eeeeeeeeee  beeeeeebbb  ebeeeebebe  beebbebbbe
 s     s s  f ffffffff     ffffs    fsffffs     s     fs sf

362         372         382         392         402
GDNVDLTVEL  IAPIAVEQGT  KFSIREGGRT  VGAGTVTEII  K
eeebebebeb  bbbbbbeebb  ebbbeeeeeb  bbbbebbebe  e
fff      s    s s        s sffff s  ss s
```

Fragment 5: 215-402

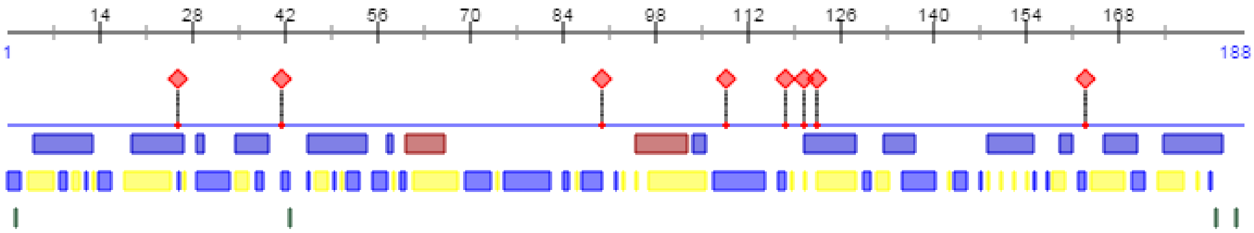


```
215         225         235         245         255
DKPFLMAVED  VFTITGRGTV  ATGKVERGQV  KLNEEVEIVG  YREEPKKTVI
eeeeebebee  bbbbbbebeb  bebebeebeb  eeeeebebbb  beeeeeeeeb
f ff sf ff   s s sfsfs   fsf ffs          f  s

265         275         285         295         305
TGIEMFNKNL  QTAMAGDNAG  VLLRGVDRKD  IERGQVIAKP  KTIIPHTKFK
ebbebbeebb  eebeeeeebb  bbbebbeeee  beebebbeee  eeeeeeeebe
f  fssff    f    ffff s  ssfs         fs s   f       f

315         325         335         345         355
AAIYALKKEE  GGRHTPFFKN  YKPQFYFRTT  DVTGGIEFEP  GREMVIPGDN
bebbbbeeee  eeeeeeebee  eeeebbbebe  eeebebebee  bbebbbeeee
   s s f f  fffffff     ffffs  fsf  fffs   s    s fs sffff

365         375         385         395
VDLTVELIAP  IAVEQGTKFS  IREGGRTVGA  GTVTEIIK
bebebebbbb  bbbeebbebb  beeeeebbbb  bbbbebee
      s      s   s  s    fffffsss   s s
```

Fragment 6: 1-215

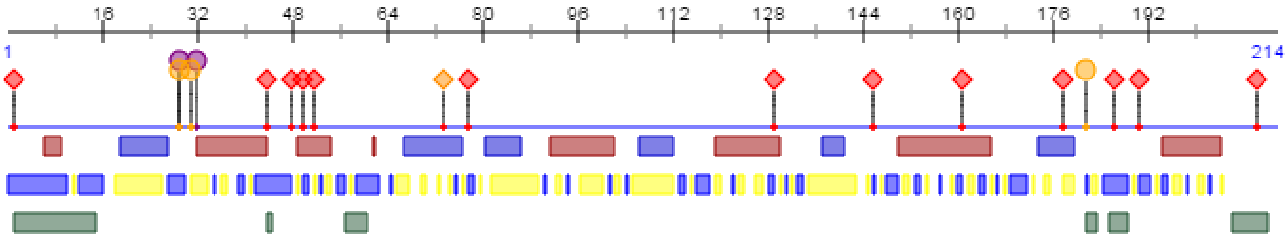


```
1           11          21          31          41
MAVVKTTGKK  DFDRSKEHIN  IGTIGHVDHG  KTTLTAAIST  VLAKRGLAEA
eebeeebbee  ebeeeeeebb  bbbbbbbeeb  ebebebbbbe  bbbeeeeeeb
      s f       f f f s  sssss ffs  fsfsfsss

51          61          71          81          91
KDYASIDAAP  EEKARGITIN  TAHIEYSTDK  RHYAHVDCPG  HADYIKNMIT
eebeebeebe  eeeeebbbbb  bbebebeeee  ebbbbbbbee  ebebbeebbb
```

```
     sf sf ff  fssss   f fs f   fssss s ff fsfs ffsss
101        111        121        131        141
GAAQMDGAIL VVAATDGPMP QTREHILLSK QVGVPKMVVF LNKIDLLEGE
bbbbbebbbb bbbbbeeeee ebeebbbbbe ebebebbbbb bbebeeeeee
sssssfs s  s  s ff ff fsffs ss  fs s    s   sf f f ff
151        161        171        181        191
EEMVDLVEVE IRELLSSYDF DGDNTPIIRG SARGALEGKP EWEAKVLELM
eebbebbeee beebbeeeeb eeeebebbeb bbbebbeeee ebeeebeebb
 f   ssf f  f ss  f    ff  f     ss                  s
201        211
DAVDSYIDSP VREM
ebbeebbeee eeee
   f   f f  f
```

Sa$_{Ef\text{-}Tu}$ (Uniprot #: Q2G0N0)

Full length protein: 1-394

1<br>MAKEKFDRSK	11<br>EHANIGTIGH	21<br>VDHGKTTLTA	31<br>AIATVLAKNG	41<br>DSVAQSYDMI
51<br>DNAPEEKERG	61<br>ITINTSHIEY	71<br>QTDKRHYAHV	81<br>DCPGHADYVK	91<br>NMITGAAQMD
101<br>GGILVVSAAD	111<br>GPMPQTREHI	121<br>LLSRNVGVPA	131<br>LVVFLNKVDM	141<br>VDDEELLELV
151<br>EMEVRDLLSE	161<br>YDFPGDDVPV	171<br>IAGSALKALE	181<br>GDAQYEEKIL	191<br>ELMEAVDTYI
201<br>PTPERDSDKP	211<br>FMMPVEDVFS	221<br>ITGRGTVATG	231<br>RVERGQIKVG	241<br>EEVEIIGLHD
251<br>TSKTTVTGVE	261<br>MFRKLLDYAE	271<br>AGDNIGALLR	281<br>GVAREDVQRG	291<br>QVLAAPGSIT
301<br>PHTEFKAEVY	311<br>VLSKDEGGRH	321<br>TPFFSNYRPQ	331<br>FYFRTTDVTG	341<br>VVHLPEGTEM
351<br>VMPGDNVEMT	361<br>VELIAPIAIE	371<br>DGTRFSIREG	381<br>GRTVGSGVVT	391<br>EIIK

Fragment 3:1-266

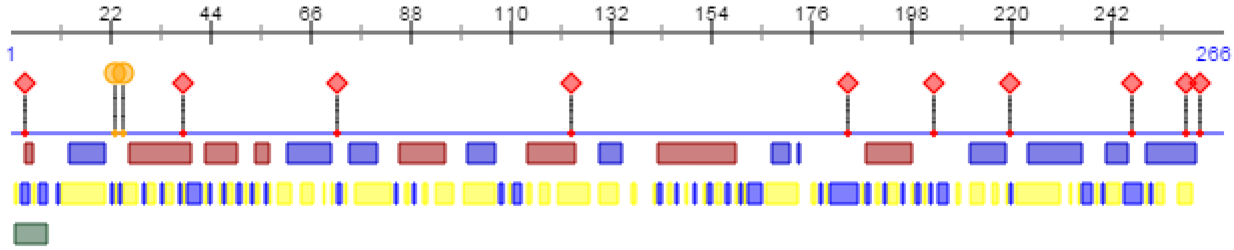

1<br>MAKEKFDRSK<br>eeeeeeeeee<br>f f f f	11<br>EHANIGTIGH<br>eebbbbbbbb<br>f s sssss	21<br>VDHGKTTLTA<br>beebebebeb<br>ffsfsfsfs	31<br>AIATVLAKNG<br>bbbebbbeee<br>ss	41<br>DSVAQSYDMI<br>eeebeebeeb<br>s
51<br>DNAPEEKERG<br>eebeeeeeeb<br>f sfff fs	61<br>ITINTSHIEY<br>bbbbbbebeb<br>sss f fs	71<br>QTDKRHYAHV<br>eeeeebbbbb<br>f fssss	81<br>DCPGHADYVK<br>bbeeebebbe<br>ssfffsfs f	91<br>NMITGAAQMD<br>ebbbbbbbbe<br>fssssssssf
101<br>GGILVVSAAD	111<br>GPMPQTREHI	121<br>LLSRNVGVPA	131<br>LVVFLNKVDM	141<br>VDDEELLELV

```
bbbbbbbbee eeeeebeebb bbbeebebeb bbbbbbebee eeeeebbebb
s s s  s f f f fsffs ss ffs s    s  sf f  fff f   ss
151        161        171        181        191
EMEVRDLLSE YDFPGDDVPV IAGSALKALE GDAQYEEKIL ELMEAVDTYI
eeebeebbee eebeeeebeb bebbbbebbe eeeeeeeebe ebbeebeeeb
f f f ss   f   ff  f      ss                 s    f

201        211        221        231        241
PTPERDSDKP FMMPVEDVFS ITGRGTVATG RVERGQIKVG EEVEIIGLHD
eeeeeeeeee bbbebeebbb bbbebebbeb ebeebebebe eebebbbbee
f f f    f s    ff    s sfsfs fs f f s          f  s
251        261
TSKTTVTGVE MFRKLL
eeeeebebbe bbeeee
      f  f ssff f
```

Fragment 4: 1-192

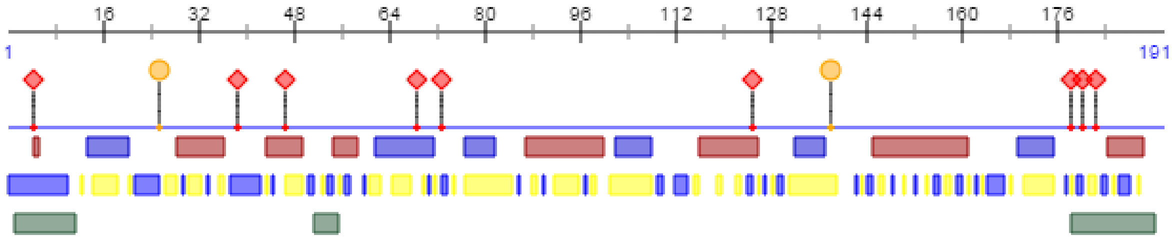


```
1          11         21         31         41
MAKEKFDRSK EHANIGTIGH VDHGKTTLTA AIATVLAKNG DSVAQSYDMI
eeeeeeeeee eebbbbbbbb beebebebeb bbbebbbeee eeebeebeeb
f f    f f  f s sssss  ffsfsfsfs ss                  s 51
                   61         71         81         91
DNAPEEKERG ITINTSHIEY QTDKRHYAHV DCPGHADYVK NMITGAAQMD
eebeeeeeeb bbbbbbebeb eeeeebbbbb bbeeebebbe ebbbbbbbbe
f sfff  fs sss    f fs  f  fssss  s fffsfs f fssssssssf101
                  111        121        131        141
GGILVVSAAD GPMPQTREHI LLSRNVGVPA LVVFLNKVDM VDDEELLELV
bbbbbbbbee eeeeebeebb bbbeebebeb bbbbbbebee eeeeebbebb
s s s  s f f fffsffs ss   fsfs     s  sf f  f f f   ss151
                  161        171        181        192
EMEVRDLLSE YDFPGDDVPV IAGSALKALE GDAQYEEKIL EL
eeebeebbee eebeeeebeb bebbbbebbe eeeeeeeeee ee
f f f ss   f   ff  f      ss
```

Fragment 5: 193-394

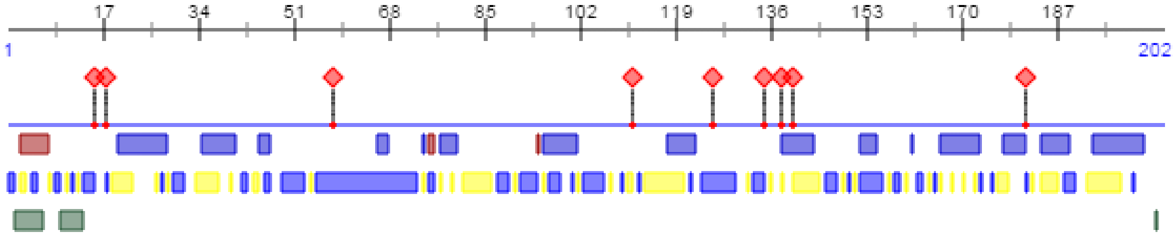


```
193         203         213         223         233
MEAVDTYIPT  PERDSDKPFM  MPVEDVFSIT  GRGTVATGRV  ERGQIKVGEE
eeebeeebee  eeeeeeeebb  bebeebbbbb  bebebbebeb  eebebebbee
    f   f   f f     s    f ff s s   sfsfs fsf   f s

243         253         263         273         283
VEIIGLHDTS  KTTVTGVEMF  RKLLDYAEAG  DNIGALLRGV  AREDVQRGQV
bebbbbeeee  eeebebbebb  eebbeebeee  eebebbbebb  eeeebeebeb
    s           f  fss  ff  f   ff  ff f s fs         fs s

293         303         313         323         333
LAAPGSITPH  TEFKAEVYVL  SKDEGGRHTP  FFSNYRPQFY  FRTTDVTGVV
beeeeebeee  eebebebbbb  eeeeeeeeee  ebeeeeeebb  bebeeeebeb
   f    ff    s      s   f fffffff  f   ffffss   fsffffs

343         353         363         373         383
HLPEGTEMVM  PGDNVEMTVE  LIAPIAIEDG  TRFSIREGGR  TVGSGVVTEI
ebeebbebbb  eeeebebebe  bbbbbbbeeb  bebbbeeeee  bbbbbbbbeb
 s    fs s  ffff             s        s sffff   sss s

393
IK
ee
```

Fragment 6: 104-192

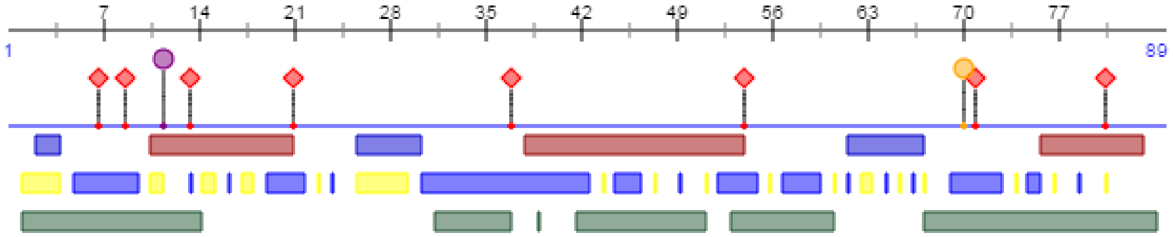


```
104         114         124         134         144
LVVSAADGPM  PQTREHILLS  RNVGVPALVV  FLNKVDMVDD  EELLELVEME
eebbbeeeee  eebeebbbbb  eebebebbbb  bbbebeeeee  eebbebbeee
ff  s ff f   fs fssss    fsfs    s    sf f f f   f   ssf f
```

154 164 174 184

VRDLLSEYDF PGDDVPVIAG SALKALEGDA QYEEKILEL

beebbeeeeb eeeebebbeb bbbebbeeee ebeeebeee

f s ff f ss s f f

Fragment 7: 214-394

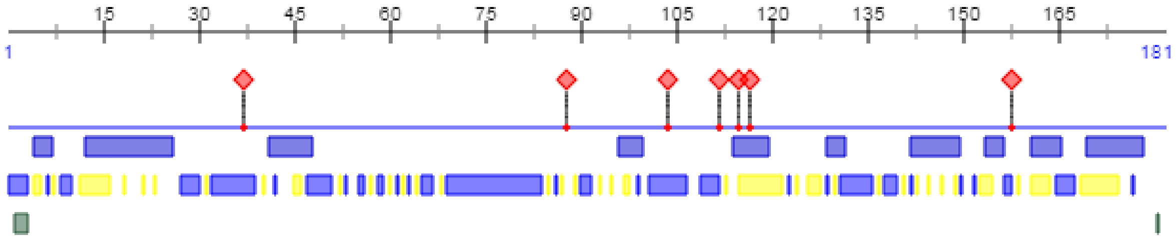

214 224 234 244 254

PVEDVFSITG RGTVATGRVE RGQIKVGEEV EIIGLHDTSK TTVTGVEMFR

eeeebbbbbb ebebbebebe ebebebbeeb ebbbbeeeee eebebbebbe

f ff s s s fsfs fsf f s s f fssf

264 274 284 294 304

KLLDYAEAGD NIGALLRGVA REDVQRGQVL AAPGSITPHT EFKAEVYVLS

ebbeeeeeee ebbbbbebbe eeebeebebb eeeeebeeee ebebebbbbe

f f fff f s s fs fs s f ff s s s 31

4 324 334 344 354

KDEGGRHTPF FSNYRPQFYF RTTDVTGVVH LPEGTEMVMP GDNVEMTVEL

eeeeeeeeee beeeeeebbb ebeeeebebe beebbebbbe eeebebebeb

f ffffffff ffffs fsff fs s fs sf fff

364 374 384 394

IAPIAIEDGT RFSIREGGRT VGSGVVTEII K

bbbbbbeebb ebbbeeeeeb bbbbbbbebe e

s s ffff s ss s

Fragment 9: 214-292

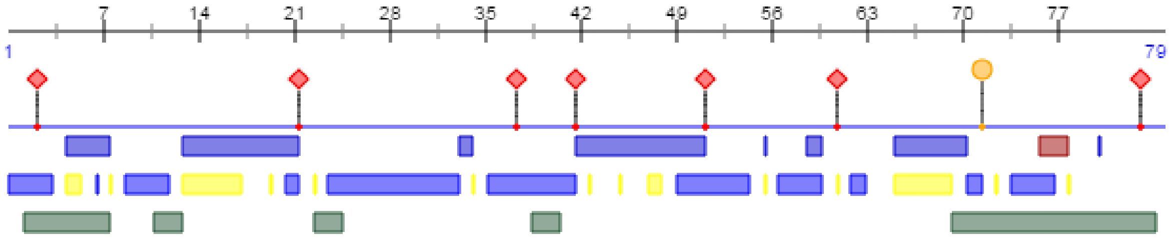

214 224 234 244 254

PVEDVFSITG RGTVATGRVE RGQIKVGEEV EIIGLHDTSK TTVTGVEMFR

eeeebbbbbb ebebbbbebe ebebebbeeb ebbbbeeeee eebebbebbe

f ff s s s fsfs ssf f s f s fs fss

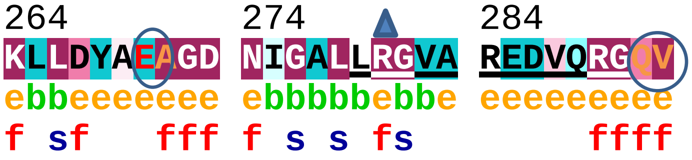
264 274 284
KLLDYAEAGD NIGALLLRGVA REDVQRGQV
ebbeeeeeeee ebbbbbebbe eeeeeeeeee
f sf fff f s s fs ffff

Will be presented as “Supplementary file S10- Experimental section”

S10. Supplementary Materials 3: Experimental Section

S10.1. Host and human proteins used in binding assays

Host proteins used for affinity chromatography include: purified fibronectin (Code: 341635) and plasminogen (Code: 528175) from human plasma supplied by Merck Millipore; plasminogen from human plasma (Code: P7999) bovine actin (Code: A3653) and fetuin (Code: F3004) supplied by Sigma.

Human proteins used for ELISA include: plasminogen (Code: P7999), lactoferrin (Code: L1294), laminin (Code: L6274), vitronectin (Code: SRP3186), plasma fibrinogen (Code: F3879) and plasma fibronectin (Code: 11051407001) supplied by Sigma.

S10.2. Peptide search parameters

Files were searched against the MSPnr100 database [151] with the following parameters. Fixed modifications: none. Variable modifications: propionamide, oxidized methionine, deamidation. Enzyme: semi-trypsin. Number of allowed missed cleavages: 3. Peptide mass tolerance: 100 ppm. MS/MS mass tolerance: 0.2 Da. Charge state: 2+, 3+ and 4+. For samples collected from the ‘Biotinylation enrichment of surface proteins’, ‘Avidin purification of A549 interacting proteins’ and ‘Avidin purification of PK-15 interacting proteins’, variable modifications also included NHS-LC-Biotin (K) and NHS-LC-Biotin (N-term). ‘Avidin purification of A549 interacting proteins’ was also searched against *homo sapiens* entries in MSPnr100 to identify biotinylated surface A549 proteins. ‘Avidin purification of PK-15 interacting proteins’ was also searched against *sus scrofa* entries in MSPnr100 to identify biotinylated surface PK-15 proteins.

S. aureus proteins were also searched against a *S. aureus* NCTC 8325 database derived from the published genome.

S10.3. Expression and purification of rMpn$_{Ef\text{-}Tu}$

Expression and purification of rMpn$_{Ef\text{-}Tu}$ was performed in one of two methods.

The first method was performed as described in [100]. In brief, the *M. pneumoniae tuf* gene (*MPN_665*) was amplified and cloned with a N-terminal hexahistadine tail into the plasmid vector pET30 (Merck Millipore) containing a kanamycin resistance gene. The recombinant construct was transformed in to BL21-DE3 competent *E. coli* cells (Merck Millipore), induced with 1 mM isopropyl-β-D-thiogalactosidase (IPTG, Roth) and purified under denaturing conditions with immobilized metal affinity chromatography Ni^{2+}-charged resin (Qiagen, Hilden, Germany) as described by the manufacturer. Elutions were concentrated using a 30 kDa Vivaspin centrifugal device (Sartorius, Göttingen, Germany). Recombinant protein was assayed and stored at -20°C. rMpn$_{Ef\text{-}Tu}$ was used to produce guinea pig antiserum as reported [100].

The second method was performed as described in [88] with modifications. In brief, the *M. pneumoniae tuf* gene (*MPN_665*) was synthesized with an N-terminal hexahistadine tail and cloned by Blue Heron Biotech (WA, USA) into a plasmid vector (PS100030) with an ampicillin resistance gene. The recombinant construct was transformed in to BL21-DE3 competent *E. coli* cells (Bioline, Eveleigh, Australia), induced with 1 mM isopropyl-β-D-thiogalactosidase (IPTG, Bioline) and purified under denaturing conditions with Profinity immobilized metal affinity chromatography Ni^{2+}-charged resin (Bio-Rad, Gladesville, Australia). BL21cells were lysed in 8 M Urea, 100 mM Na_2HCO_3, 10 mM Tris-HCl, pH 8 with 6 rounds of sonication for 30 seconds on ice. Following centrifugation, the supernatant was added to Ni2+ resin overnight at 4°C. The

resin was then loaded onto a column, washed four times with 5 ml 8 M Urea, 100 mM Na_2HCO_3, 10 mM Tris-HCl, pH 6.3 and a 2-step elution: three times with 5 ml 8 M Urea, 100 mM Na_2HCO_3, 10 mM Tris-HCl, pH 5.9 and twice with 10 ml 8 M Urea, 100 mM Na_2HCO_3, 10 mM Tris-HCl, pH 4.5**.** Washes and elutions were monitored by SDS-PAGE. Elutions were concentrated using a 10 kDa Microsep™ centrifugal device (Pall, Port Washington, NY) and dialysed into PBS, 0.5% Tween 20 with 10,000 MWCO SnakeSkin® Dialysis Tubing (Thermo Fisher Scientific) at 4°C. Recombinant protein was assayed and stored at 4°C.

S10.4 LC-MS/MS of dimethyl labelled proteins

S10.4.1. LC-MS/MS (Sciex 5600) of dimethyl labelled proteins

Peptides from dimethyl labelled proteins described in section 1.16.1 were separated by nanoLC using an Ultimate nanoRSLC UPLC and autosampler system (Dionex, Amsterdam, Netherlands). Samples (2.5 µl) were concentrated and desalted onto a micro C18 precolumn (300 µm x 5 mm, Dionex) with H_2O:CH_3CN (98:2, 0.1 % TFA) at 15 µl/min. After a 4 min wash the pre-column was switched (Valco 10 port UPLC valve, Valco, Houston, TX) into line with a fritless nano column (75µ x ~15cm) containing C18AQ media (1.9µ, 120 Å Dr Maisch, Ammerbuch-Entringen Germany). Peptides were eluted using a linear gradient of H_2O:CH_3CN (98:2, 0.1 % formic acid) to H_2O:CH_3CN (64:36, 0.1 % formic acid) at 200 nl/min over 240 min. High voltage 2000 V was applied to low volume Titanium union (Valco) with the tip positioned ~ 0.5 cm from the curtain plate (T=150°C) of a 5600^+ mass spectrometer (Sciex, Toronto, Canada). Positive ions were generated by electrospray and the 5600^+ operated in information dependent acquisition mode (IDA).

A survey scan m/z 350-1750 was acquired (PWHH resolution ~30,000, 0.25 sec acquisition time) with autocalibration enabled (at ~6 hr intervals). Up to the 10 most abundant ions (>300 counts) with charge states > +2 and <+5 were sequentially isolated (width m/z ~3) and fragmented by CID with an optimal CE chosen based on m/z (product ion spectra were acquired at a resolution ~20,000 PWHH in 0.15 sec). M/z ratios selected for MS/MS were dynamically excluded for 30 or 45 seconds.

Peak lists were generated using Mascot Daemon/Mascot Distiller (Matrix Science, London, England) or ProteinPilot (Sciex, v4.5) using default parameters, and submitted to the database search program Mascot (version 2.5.1, Matrix Science). Search parameters were: Precursor tolerance 10 ppm and product ion tolerances ± 0.05 Da; oxidation (M), deamidation (NQ), propionamide (C), Dimethyl (K), Dimethyl (N-term) specified as variable modifications; enzyme specificity was semi-ArgC; 1 missed cleavage was possible and the non-redundant protein database from NCBI (Jan 2015) searched.

S10.4.2. LC-MS/MS (Thermo Scientific Q Exactive™) of dimethyl labelled proteins

Peptides from dimethyl labelled proteins described in section 1.18.1 were separated by nanoLC using an Ultimate nanoRSLC UPLC and autosampler system (Dionex, Amsterdam, Netherlands). Samples (2.5 µl) were concentrated and desalted onto a micro C18 precolumn (300 µm x 5 mm, Dionex) with H_2O:CH_3CN (98:2, 0.1 % TFA) at 15 µl/min. After a 4 min wash the pre-column was switched (Valco 10 port UPLC valve, Valco, Houston, TX) into line with a fritless nano column (75µ x ~35cm) containing C18AQ media (1.9µ, 120 Å Dr Maisch, Ammerbuch-Entringen Germany). Peptides were eluted using a linear gradient of H_2O:CH_3CN (98:2, 0.1 % formic acid) to H_2O:CH_3CN (64:36, 0.1 % formic acid) at 200 nl/min over 30 or

240 min. High voltage 2000 V was applied to low volume Titanium union (Valco) with the column oven heated to 45°C (Sonation, Biberach, Germany) and the tip positioned ~ 0.5 cm from the heated capillary (T=300°C) of a QExactive Plus mass spectrometer (Thermo Fisher Scientific, Bremen, Germany). Positive ions were generated by electrospray and the QExactive operated in data dependent acquisition mode (DDA).

A survey scan m/z 350-1750 was acquired (resolution = 70,000 at m/z 200, with an AGC target value of 10^6 ions) and lockmass was enabled (m/z 445.12003) Up to the 10 most abundant ions (>80,000 counts, underfill ratio 10%) with charge states > +2 and <+7 were sequentially isolated (width m/z 2.5) and fragmented by HCD (NCE = 30) with a AGC target of 10^5 ions (resolution = 17,500 at m/z 200). M/z ratios selected for MS/MS were dynamically excluded for 30 or 45 seconds.

Peak lists were generated using Mascot Daemon/Mascot Distiller (Matrix Science, London, England) or Proteome Discoverer (Thermo Fisher Scientific, v1.4) using default parameters, and submitted to the database search program Mascot (version 2.5.1, Matrix Science). Search parameters were: Precursor tolerance 4 ppm and product ion tolerances ± 0.05 Da; oxidation (M), deamidation (NQ), propionamide (C), Dimethyl (K), Dimethyl (N-term) specified as variable modifications; enzyme specificity was semi-ArgC; 1 missed cleavage was possible and the non-redundant protein database from NCBI (Jan 2015) searched.

References

149 Yachdav, G. et al. PredictProtein--an open resource for online prediction of protein structural and functional features. Nucleic acids research 42, W337-343, doi:10.1093/nar/gku366 (2014).

150 Hönigschmid, P. Improvement of DNA- and RNA protein binding prediction., Technische Universität München, (2012).

151 Perkins, D. N., Pappin, D. J. C., Creasy, D. M. & Cottrell, J. S. Probability-based protein identification by searching sequence databases using mass spectrometry data. Electrophoresis 20, 3551-3567, doi:10.1002/(SICI)1522-2683(19991201)20:18<3551::AID-ELPS3551>3.0.CO;2-2 (1999).
